# Deletion of *Hsd11b1* suppresses caloric restriction-induced bone marrow adiposity in male but not female mice

**DOI:** 10.1530/JOE-24-0072

**Published:** 2024-06-24

**Authors:** Andrea Lovdel, Karla J Suchacki, Fiona Roberts, Richard J Sulston, Robert J Wallace, Benjamin J Thomas, Rachel M B Bell, Iris Pruñonosa Cervera, Gavin J Macpherson, Nicholas M Morton, Natalie Z M Homer, Karen E Chapman, William P Cawthorn

**Affiliations:** 1 University/BHF Centre for Cardiovascular Science, The University of Edinburgh, The Queen’s Medical Research Institute, Edinburgh BioQuarter, Edinburgh, UK; 2 Department of Orthopaedics, The University of Edinburgh, Edinburgh, UK; 3 Department of Orthopaedic Surgery, Royal Infirmary of Edinburgh, Edinburgh, UK; 4 Centre for Systems Health and Integrated Metabolic Research, Department of Biosciences, School of Science and Technology, Nottingham Trent University, Nottingham, UK

**Keywords:** bone marrow adipose tissue, bone, caloric restriction, 11β-HSD1, glucocorticoids, progesterone, sex differences

## Abstract

Bone marrow adipose tissue (BMAT) comprises >10% of total adipose mass in healthy humans. It increases in diverse conditions, including ageing, obesity, osteoporosis, glucocorticoid therapy, and notably, during caloric restriction (CR). BMAT potentially influences skeletal, metabolic, and immune functions, but the mechanisms of BMAT expansion remain poorly understood. Our hypothesis is that, during CR, excessive glucocorticoid activity drives BMAT expansion. The enzyme 11β-hydroxysteroid dehydrogenase type 1 (11β-HSD1) amplifies glucocorticoid activity by catalysing intracellular regeneration of active glucocorticoids from inert 11-keto forms. Mice lacking 11β-HSD1 resist metabolic dysregulation and bone loss during exogenous glucocorticoid excess; thus, we hypothesised that 11β-HSD1 knockout mice would also resist excessive glucocorticoid action during CR, thereby restrining BMAT expansion and bone loss. To test this, we first confirmed that 11β-HSD1 is expressed in mouse and human bone marrow. We then investigated the effects of CR in male and female control and 11β-HSD1 knockout mice from 9 to 15 weeks of age. CR increased *Hsd11b1* mRNA in adipose tissue and bone marrow. Deletion of *Hsd11b1* did not alter bone or BMAT characteristics in mice fed a control diet and had little effect on tibial bone microarchitecture during CR. Notably, *Hsd11b1* deletion attenuated the CR-induced increases in BMAT and prevented increases in bone marrow corticosterone in males but not females. This was not associated with suppression of glucocorticoid target genes in bone marrow. Instead, knockout males had increased progesterone in plasma and bone marrow. Together, our findings show that knockout of 11β-HSD1 prevents CR-induced BMAT expansion in a sex-specific manner and highlights progesterone as a potential new regulator of bone marrow adiposity.

## Introduction

Bone marrow adipocytes comprise up to 70% of total bone marrow (BM) volume and over 10% of total adipose mass in healthy adult humans, collectively forming an integrated tissue referred to as bone marrow adipose tissue (BMAT) (Bravenboer *et al.* 2020, Cawthorn 2020). Bone marrow adipocytes further accumulate in diverse conditions, including ageing, obesity, type 2 diabetes, osteoporosis, chronic kidney disease, and in iatrogenic contexts such as chronic glucocorticoid treatment, cancer chemotherapy, or radiotherapy (Cawthorn 2020). The mechanisms by which BMAT is formed and accumulates are largely unknown. Similarly, the role of BMAT remains unclear, though it has been implicated in the physiological and pathological regulation of various processes, including metabolic homeostasis, haematopoiesis, skeletal remodelling, and progression of haematological tumours and skeletal metastases (Attané *et al.* 2020, Cawthorn 2020, Morris *et al.* 2020, Pham *et al.* 2020, Suchacki *et al.* 2020, Heydt *et al.* 2021, Li *et al.* 2022*a*, *b*, Austin *et al.* 2023). This potential importance of BMAT in health and disease has motivated a surge of research into BM adiposity over the past decade. Nevertheless, there remains a critical need to better understand BMAT formation and function.

**Table 1:** Key resources.

Reagent or resource	Source	Identifier
Chemicals		
Osmium tetroxide	Agar Scientific (Stansted, UK)	AGR1022
RiboZol	AMRESCO (Cleveland, OH, USA)	N580
Experimental models**:**organisms/strains		
Mouse: C57BL/6JOlaHsd	Karen Chapman (Verma *et al.* 2018)	Not applicable
Software and programmes		
CT Analyzer	Bruker (Kontich, Belgium)	v1.16.4.1
ImageJ	NIH (Bethesda, MD, USA)	V1.32
Prism	GraphPad Software, LLC	V10.0.2
Other		
OneTouch Verio Glucometer	OneTouch (LifeScan, Milpitas, CA, USA)	User’s manual^a^
Faxitron	Bioptics (Tucson, AZ, USA)	43855D
Phenomenex Kinetex C8 HPLC column	Phenomenex (Torrance, CA, USA)	00D-4499-AN

^a^OneTouch Verio Glucometer user manual: https://www.onetouch.com/sites/onetouch_us/files/06908603a_vro_ob_us_en_r1_full_web_v2_fvid177812.pdf

One striking observation is that, in contrast to other adipose depots, BMAT accumulates in conditions of caloric restriction (CR), including anorexia nervosa or short-term fasting in humans and in animal models of CR (Cawthorn 2020, Fazeli *et al.* 2021). CR has garnered extensive therapeutic interest because of its ability to extend lifespan and reduce the risk of age-related diseases in numerous species, ranging from yeast to primates (Speakman & Mitchell 2011). However, CR can also promote bone loss (Villareal *et al.* 2015) and increased susceptibility to infections (Speakman & Mitchell 2011). Aside from these clinical implications, many effects of CR represent fundamental evolutionary adaptations that aid survival during times of starvation (Speakman & Mitchell 2011). Therefore, understanding BMAT formation and function during CR could yield new insights into fundamental biology and mechanisms of healthy ageing.

Our previous studies suggest that glucocorticoid excess may contribute to BMAT expansion during CR. We have shown that both BMAT and circulating glucocorticoids increase during CR in mice, whereas neither of these increases occurs during CR in rabbits (Cawthorn *et al.* 2016). This implies that the endogenous increase in circulating glucocorticoids is associated with BMAT expansion during CR. BMAT also increases in other conditions of glucocorticoid excess, including exogenous treatment and in Cushing’s disease (Vande Berg *et al.* 1999, Li *et al.* 2013*a*, *b*), while remission from Cushing’s disease leads to decreased BM adiposity (Geer *et al.* 2012). Thus, glucocorticoid excess is sufficient to increase BM adiposity, and this is reversed with the restoration of normal glucocorticoid activity. Based on this, our hypothesis is that increased glucocorticoid action underlies CR-induced BMAT expansion and that suppression of endogenous glucocorticoid action will restrict BMAT expansion during CR.

Intracellular glucocorticoid exposure is determined not only by circulating concentrations but also by the intracellular regeneration of active glucocorticoids from inert 11-keto forms; this reaction is driven by the enzyme 11β-hydroxysteroid dehydrogenase type 1 (11β-HSD1) (Tomlinson *et al.* 2004). To determine the function of this enzyme *in vivo*, an 11β-HSD1 knockout (KO) mouse model was first created in 1997 by introducing a neomycin-resistance cassette intended to replace exons 3 and 4 of the *Hsd11b1* gene (Kotelevtsev *et al.* 1997). Justesen *et al.* reported that these KO mice lack BM adipocytes (Justesen *et al.* 2004); however, we subsequently showed that this is not the case, with BM adipocytes being readily detectable in carpal and tarsal joints of these mice (Coutinho *et al.* 2012). Moreover, although this mouse model was at first considered a global KO, our data show that it continues to express *Hsd11b1* at low levels in some tissues, including the lung and kidney (Yang 2010); hence, this original KO line is more accurately described as a hypomorphic model (*Hsd11b1^hypo^*), rather than being a total *Hsd11b1* KO (*Hsd11b1^null^*).

This limitation has since been overcome by the creation of two improved, total KO mouse lines, in which Cre-lox technology was used to delete either exon 3 (Verma *et al.* 2018) or exon 5 (Semjonous *et al.* 2011) of the *Hsd11b1* gene, resulting in a total lack of *Hsd11b1* expression. It remains unknown if BM adiposity is altered in these full KO mice. A critical finding for our present study is that total *Hsd11b1* KO mice resist metabolic dysregulation and trabecular bone loss caused by exogenous corticosterone excess (Morgan *et al.* 2014, Fenton *et al.* 2019). Thus, we hypothesised that *Hsd11b1* KO mice would also resist excessive glucocorticoid action during CR, thereby restraining BMAT expansion and bone loss. Herein, we first confirmed that 11β-HSD1 transcripts are expressed within the BM of mice and humans. We then addressed this hypothesis by studying the effects of CR in male and female total *Hsd11b1* KO mice and wild-type counterparts. Our results shed new light on the mechanisms through which CR impacts glucocorticoid action, BMAT, and bone biology.

## Materials and methods

### Key resources

The key resources used in this study are described in Table 1.

### Animals

Mouse studies were approved by the University of Edinburgh Animal Welfare and Ethical Review Board and conducted under UK Home Office licences. Global *Hsd11b1* ^Del1/Del1^ (KO) mice on a C57BL/6JOlaHsd genetic background were generated by Cre-LoxP deletion of exon 3 (Vandermosten *et al.* 2017, Zhang *et al.* 2017, Verma *et al.* 2018). Heterozygotes were mated to generate experimental KO mice and control (WT) mice; the latter retained both WT *Hsd11b1* alleles. Littermate controls were used throughout. Genotyping was done by Transnetyx (Cordova, TN, USA). Mice were housed at 22–23ºC on a 12 h light:12 h dark cycle in a specific-pathogen-free facility with free access to water and food, as indicated. Table 2 shows details of the mouse cohorts, the groups being compared, and the experimental unit. The exact number of mice used is stated in the figure legends. Sample sizes were determined by power calculations (G*Power software), with effect sizes based on previous data for CR-induced BMAT expansion (the primary outcome) (Cawthorn *et al.* 2014, 2016). Randomisation, blinding, and exclusion of mice from final analyses were as described previously (Suchacki *et al.* 2023). Fourteen mouse tibiae (six WT CR males, one KO CR male, five WT CR females, and two KO CR females) were excluded from the micro-computed tomography (µCT) analysis of proximal BMAT because of ruptures in the proximal tibia during processing, which impairs BMAT quantification (Sulston *et al.* 2016).

**Table 2:** Summary of CR protocol for each group of mice. Because mice are singly housed, each mouse represents an independent experimental unit.

	1-week CR	6-week CR
Age at single housing	8 weeks	8 weeks
Age at start of CR	9 weeks	9 weeks
Time of feeding	09:00–10:00	09:00–10:00
Duration of CR	1 week	6 weeks
Fasting status at necropsy	Random-fed (AL mice) or fasted ~21 h (CR mice)	Fasted ~12 h (for both AL and CR mice)
Group sizes	Male WT (*n* = 16), female WT (*n* = 11), male KO (*n* = 10), female KO (*n* = 17)	Male WT (*n* = 18), female WT (*n* = 21), male KO (*n* = 16), female KO (*n* = 14)
Related data	Figs 3C, D, E, F, G, H, 7 and 8	Figs 2, 3A, 3B, 4, 5 and6; Supplementary Fig. 4
Experimental unit	Single mouse (for both 1-week CR and 6-week CR cohorts)	
Groups compared	AL vs CR (within sex); WT vs KO (within diet); male vs female (within diet); male vs female (within genotype); and interactions of the three variables (diet, genotype, and sex)	

CR studies, including the *ad libitum* (AL) and CR dietary protocols, analysis of body composition, and endpoint tissue processing, were done as described previously (Suchacki *et al.* 2023). CR mice were fed a micronutrient-enriched diet to prevent micronutrient deficiency.

### Human subjects and tissue isolation

Bone marrow and subcutaneous white adipose tissue biopsies were obtained with written informed consent from human donors undergoing hip-replacement surgery. The study was approved by the South East Scotland Research Ethics Committee (REC) with ethics number 10/S1102/39. Donor characteristics are described in Table 3. BM and WAT were isolated from patients undergoing hip replacement surgery, as described previously (Suchacki *et al.* 2020, Lucas *et al.* 2021).

**Table 3:** Human subject characteristics. Age and BMI are mean ± s.d.

	Age	BMI	Diabetic (*n*)	Osteoporotic (*n*)
Both sexes (*n* = 16)	68.1 ± 9.5	30.0 ± 3.4	0	2
Males (*n* = 11)	68.6 ± 10.0	29.0 ± 2.5	0	1
Females (*n* = 5)	67.2 ± 9.3	32.2 ± 5.9	0	1

### Histology and histomorphometry

Fixed murine WAT and decalcified bones (14% EDTA for 14 days) were paraffin-embedded, sectioned, H&E stained, and analysed for adipocyte size distribution as described previously (Suchacki *et al.* 2023).

### X-ray analysis of bone density and length

WT and *Hsd11b1^−/−^* humeri, femurs, tibiae, and vertebrae were X-rayed using a Faxitron 43855D cabinet (Bioptics, Tucson, AZ, USA). Exposure was set to 15 s at 21 kV. Bones were analysed using ImageJ v1.32 (NIH, Bethesda, MD, USA).

### Bone analysis by micro-computed tomography scanning

Tibiae were fixed in 10% formalin at 4°C for 2 days. For bone architecture, calcified tibiae were embedded in 1% agarose (w/v in deionised water) in 30 mL universal tubes: each tube contained two layers of 5–6 tibiae arranged in parallel (10–12 per tube). Tibiae then underwent µCT scanning as described below. For BMAT measurements, tibiae were decalcified, stained with osmium tetroxide (1% w/v; Agar Scientific, Stansted, UK) for 48 h at room temperature, washed in Sorensen’s phosphate buffer (81 mM KH_2_PO_4_, 19 mM Na_2_HPO_4_·7H_2_O, pH 7.4), and then embedded in 1% agarose for µCT scanning, as for calcified bones.

For µCT, 30 mL tubes were mounted in a Skyscan 1172 desktop µCT scanner (Bruker, Kontich, Belgium) and scanned through 360°, using a step of 0.40° between exposures. An isotropic voxel resolution of 6 μm was obtained for calcified bones and 12.05 µm for osmium-stained bones using a 54 kV source voltage, 185 µA source current, and a filter exposure time of 1767 ms for calcified bones and 885 ms for osmium-stained bones. Scans were optimised using a 0.5 mm aluminium filter and two-frame averaging (calcified bones) or four-frame averaging (osmium-stained bones). Scans were reconstructed using NRecon v1.7.3.0 and bone morphology and BMAT was then quantified using CT Analyser v1.16.4.1 (Bruker).

Trabecular microarchitecture was assessed within the proximal metaphysis (slices 16–167 below the proximal growth plate), and cortical parameters within the proximal diaphysis (slices 500–583 below the proximal growth plate). Total marrow volume (Ma.V), excluding fibulae, was measured for proximal (proximal growth plate to the tibia–fibula junction) and distal (tibia–fibula junction to the bottom of the bone) regions of calcified tibiae. BMAT volume in these regions was then determined in osmium-stained bones. The threshold for BMAT quantification was set to 70–255 to exclude background noise. BMAT volume in distal, proximal, or total tibiae (distal + proximal) was then quantified as absolute volume (mm^3^) or as a % of the corresponding Ma.V for each region.

### RNA isolation, reverse transcription, and qPCR

For mouse and human tissues, RNA isolation, reverse transcription, primer design/validation, and qPCR were done as described previously (Suchacki *et al.* 2020). To isolate BM from fresh or frozen mouse tibiae, a razor blade was used to cut off the proximal and distal ends of each tibia and to remove the bottom of a 0.5 mL microtube. Each cut tibia was then placed into a cut 0.5 mL microtube, which was inserted into a 2 mL microtube and centrifuged at 8000 ***g*** for 1 min at 4ºC. This flushed the BM from the tibia into the bottom of each 2 mL microtube. Tibial BM was homogenised in 500 µL RiboZol reagent (AMRESCO LLC, Cleveland, OH, USA). RNA was quantified using a NanoDrop spectrophotometer (Thermo Scientific), and integrity was confirmed by gel electrophoresis analysis of 18S and 28S rRNA.

For human samples, expression of *HSD11B1* was normalised to the average expression of 18S rRNA (human gene, *RNA18SN5*) and *TBP*. For mouse samples, expression of target genes was normalised to the geometric mean of housekeeping genes suitable for each tissue (Vandesompele *et al.* 2002), based on these genes not showing any regulation under the experimental conditions tested; figure legends describe the specific housekeeping genes used. For each transcript, mRNA levels are expressed relative to the group with the highest levels. TaqMan assays (Thermo Fisher) were used to analyse *Hsd11b2* (cat. no. Mm01251104_m1), *Fkbp5* (cat. no. Mm00487406_m1), *Gapdh* (cat. no. Mm99999915_g1), *Tsc22d3* (also known as *Gilz;* cat. no. Mm00726417_s1), and *Per1* (cat. no. Mm00501813_m1) in mouse tissues. All other primers are described in Table 4.

**Table 4:** Primers used for Sybr Green qPCR.

Species	Transcript	Forward primer (5′–3′)	Reverse primer (5′–3′)
*H. sapiens*	*HSD11B1*	GTCCAAACCGGTGACTTTCT	GGCAGCAACCATTGGATAAG
*H. sapiens*	*TBP*	CCGGCTGTTTAACTTCGCTTC	CAAGAAACAGTGATGCTGGGT
*H. sapiens*	*RNA18SN5*	CGATGCTCTTAGCTGAGTGT	GGTCCAAGAATTTCACCTCT
*M. musculus*	*Actb*	CACTGTCGAGTCGCGTCC	TCATCCATGGCGAACTGGTG
*M. musculus*	*Hprt*	TCATTATGCCGAGGATTTGGA	GCACACAGAGGGCCACAAT
*M. musculus*	*Hsd11b1*	AGACCAGAAATGCTCCAGGG	ATAAGCATGTCCAGTCCGCC
*M. musculus*	*Ppia*	CACCGTGTTCTTCGACATCA	CAGTGCTCAGAGCTCGAAAGT
*M. musculus*	*Rn18s*	CGATGCTCTTAGCTGAGTGT	GGTCCAAGAATTTCACCTCT
*M. musculus*	*Tbp*	ACCTTATGCTCAGGGCTTGG	GCCGTAAGGCATCATTGGAC

### Liquid chromatography–tandem mass spectrometry for steroid analysis in mouse plasma and BM

LC-MS/MS was used to quantify the steroid hormones 11-dehydrocorticosterone (11-DHC), corticosterone (CORT), testosterone, and progesterone in endpoint plasma and BM from male and female mice, using a 13-point calibration curve and isotopically labelled internal standards, as described (Lovdel *et al.* 2024). This method used supported liquid extraction (SLE) and automation to improve the precision of sample preparation while reducing the volume of sample required: 50 µL plasma and ~15 mg femoral BM were used per mouse. BM was isolated from pre-frozen femurs (as described above for RNA extraction from tibial BM), and each BM sample was homogenised in 1000 µL of acetonitrile containing 0.01% formic acid (v/v). The lower limit of quantitation (LLOQ) in bone marrow was 0.025 ng/g for 11-DHC and CORT, 0.005 ng/g for testosterone, and 0.01 ng/g for progesterone. The LLOQ in plasma was 0.025 ng/mL for 11-DHC, CORT, and 0.01 ng/mL for testosterone and progesterone. These analyses were done on 10-week-old mice, after 1 week of AL or CR diet, without fasting AL mice prior to necropsy. This is because we found that fasting AL mice (as done for our 15-week-old cohort) increased corticosterone levels and blunted detection of the CR effect (data not shown).

### Statistical analysis, data presentation, and reproducibility

Statistical analyses and data presentation were done as described previously (Suchacki *et al.* 2020, Suchacki *et al.* 2023), with further details provided in the figure legends. A *P*-value < 0.05 (after adjustment for multiple comparisons) was considered statistically significant. Units and abbreviations are reported in accordance with guidelines for BM adiposity research (Bravenboer *et al.* 2020). Where representative micrographs or µCT images are shown, figure legends describe the number of biologically independent samples that these represent.

### Data availability

All source data from which the figures are based are available on the University of Edinburgh DataShare (https://doi.org/10.7488/ds/7730).

## Results

### 11β-HSD1 mRNA expression is similar in BM and white adipose tissue and, in mice, is upregulated during CR

**Figure 1:**
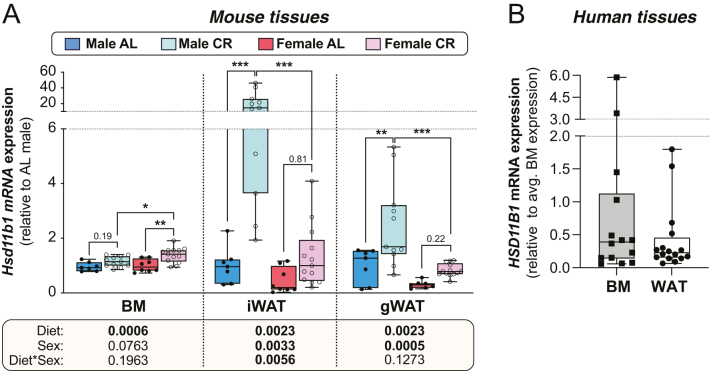
Transcripts encoding 11β-HSD1 are expressed at similar levels in BM and WAT and are increased with CR. (A) Male and female mice on a C57BL/6JOlaHsd background were fed *ad libitum* (AL) or a 30% CR diet from 9 to 15 weeks of age (0–6 weeks of CR). At necropsy (15 weeks’ old) tibial BM, iWAT, and gWAT were sampled, and expression of *Hsd11b1* was determined by qPCR. Expression is shown relative to levels in AL males after normalising to the geometric mean of the housekeeping genes *Ppia, Tbp,* and *Actb* (for BM) or *Ppia, Tbp,* and *Hprt* (for iWAT and gWAT). Box-and-whisker plots include the following numbers of mice per group: male AL,*n* = 7; female AL,*n* = 8; male CR,*n* = 11; female CR,*n* = 13. Within each tissue, significant effects of diet, sex, and diet × sex interactions were determined by two-way ANOVA, with *P* valuesshown beneath the graph. Within each tissue, significant diet effects (within each sex) or sex effects (within each diet) were determined by Fisher’s LSD test and are indicated by *P < 0.05, **P < 0.01, or ***P < 0.001. (B) *HSD11B1* expression in femoral BM or subcutaneous WAT of human donors (*n* = 16) was assessed by qPCR. Expression is shown as box-and-whisker plots relative to levels in BM after normalising to the geometric mean of the housekeeping genes *TBP* and *RN18S.* There was no significant difference between BM and WAT expression, as determined by the Wilcoxon matched-pairs signed-rank test. Source data are provided as a Source Data file.

To determine the potential for 11β-HSD1 to influence BM adiposity, we first investigated the extent of 11β-HSD1 mRNA expression within the BM and whether these changes during CR. As shown in Fig. 1A, *Hsd11b1* was readily detectable in the BM of 15-week-old wild-type AL-fed male and female mice. CR from 9 to 15 weeks of age increased *Hsd11b1* transcripts within the BM (*P,* diet = 0.0006) and this effect tended to be stronger in females than in males (Fig. 1A). We also analysed *Hsd11b1* expression in inguinal and gonadal WAT depots (iWAT and gWAT, respectively) of these mice to compare with its expression within BM. As for the BM, CR increased *Hsd11b1* expression in iWAT and gWAT, albeit to a greater extent in males than in females (Fig. 1A). The BM reportedly has very low expression of 11β-HSD2 (Thorrez *et al.* 2008), but whether this is increased in CR remains unknown. Therefore, we also measured the expression of 11β-HSD2 mRNA (*Hsd11b2)* to assess if this enzyme may also influence BM glucocorticoid exposure. The expression of *Hsd11b2* in the BM was very low, with an average qPCR cycle threshold of 36.2 ± 0.7 (mean ± s.d.) across all mice. In contrast, *Hsd11b1* expression levels were much higher, with an average qPCR cycle threshold of 25.7 ± 0.8. Moreover, unlike for *Hsd11b1*, 6 weeks of CR did not affect *Hsd11b2* expression in the BM of male or female mice (Supplementary Fig. 1, see section on supplementary materials given at the end of this article).

To determine the translational relevance of these findings, we measured *HSD11B1* expression in femoral BM and subcutaneous WAT from human donors. As shown in Fig. 1C, *HSD11B1* was expressed at similar levels in these two tissues, consistent with previous findings in mice (Thorrez *et al.* 2008). Among all tissues, WAT has relatively high expression of 11β-HSD1 (Tomlinson *et al.* 2004); thus, our findings show that, unlike 11β-HSD2, 11β-HSD1 is highly expressed within the BM and, in mice, this expression is further upregulated during CR.

### Global knockout of Hsd11b1 does not alter peripheral adiposity, bone length, bone density, or BM adiposity in young adult mice fed a normal chow diet

To investigate if *Hsd11b1* KO influences BM adiposity, we first analysed mice fed a normal chow diet. We began by determining if total *Hsd11b1* KO mice have altered peripheral adiposity, bone density, and/or skeletal development, each of which is associated with altered BM adiposity (Cawthorn 2020, Morris *et al.* 2024). To do so, we analysed KO and WT mice at 13 weeks of age. Total body mass and the masses of iWAT and gWAT were similar between KO and WT mice (Supplementary Fig. 2A and B), consistent with findings in another total *Hsd11b1* KO mouse line (Morgan *et al.* 2014). We found a small genotype–sex interaction for gWAT mass, with *Hsd11b1* KO tending to increase it in males but decrease it in females (Supplementary Fig. 2B). Consistent with this, *Hsd11b1* KO did not affect adipocyte size distribution in male iWAT or gWAT, or in female iWAT (Supplementary Fig. 2C, D, and E), but it increased the proportion of small adipocytes in female gWAT (Supplementary Fig. 2F). Together, these data show that total *Hsd11b1* KO has a small sex-specific effect on visceral adiposity, but total peripheral adiposity is grossly similar between WT and total KO mice.

We next analysed the effects on the long bones, first by using X-rays to analyse humeri, femurs, and tibiae. This revealed that, in these 13-week-old mice, femoral and tibial bone density was lower in females than in males but was unaffected by *Hsd11b1* KO, and that neither sex nor genotype influenced humeral density (Supplementary Fig. 3A and B). Similarly, the lengths of these bones were unaffected by sex or genotype (Supplementary Fig. 3C). Thus, bone density and bone length are grossly similar between WT and KO mice.

Finally, we analysed BM adiposity of these 13-week-old mice, both histologically and by using osmium tetroxide to quantify BMAT volume in the long bones (Scheller *et al.* 2014). BM adipocytes were abundant in the caudal vertebrae of both WT and KO males, and KO had no effect on BM adipocyte size (Supplementary Fig. 4A). Humeral BMAT volume was very low in both genotypes and was unaffected by sex or *Hsd11b1* KO (Supplementary Fig. 4B and C). BMAT in femurs and tibiae was more abundant than in the humeri: femoral BMAT predominated around the proximal and distal epiphyses, whereas tibial BMAT was greatest below the tibia–fibula junction, in the distal region corresponding to ‘constitutive’ BMAT (Scheller *et al.* 2015) (Supplementary Fig. 4D, E, F, and G). The volume of femoral BMAT and distal tibial BMAT was greater in females than males, while ‘regulated’ BMAT volume in the proximal tibia (Scheller *et al.* 2015) was similar between the sexes. These sex differences were not expected but show that BM adiposity differs between males and females in a site-dependent manner, as we recently reported in humans (Morris *et al.* 2024). However, as for humeri and caudal vertebrae, *Hsd11b1* KO did not affect femoral or tibial BMAT volume (Supplementary Fig. 4D, E, F, and G).

### Effects of Hsd11b1 KO on body mass and body composition during CR

These data show that *Hsd11b1* KO does not impact BMAT or bones of mice fed a normal diet. Because *Hsd11b1* is upregulated in WAT and BM during CR (Fig. 1A), and CR increases systemic glucocorticoid exposure (Cawthorn *et al.* 2016), we next investigated if *Hsd11b1* KO alters the metabolic, endocrine, and skeletal effects of CR. WT and KO male and female mice were fed *ad libitum* or a CR diet (70% of daily AL intake) from 9 to 15 weeks of age. Using qPCR, we found that *Hsd11b1* mRNA was undetectable in iWAT, gWAT and BM of KO mice, confirming the total KO of *Hsd11b1* (data not shown). To determine if KO influenced *Hsd11b2* expression*,* we measured *Hsd11b2* transcripts within the BM of WT and KO mice after 1 week of AL or CR diet. This duration of CR caused a small but significant decrease in *Hsd11b2* mRNA; however, *Hsd11b1* KO did not alter the very low levels of *Hsd11b2* expression within the BM, irrespective of sex or diet (Supplementary Fig. 5).

**Figure 2:**
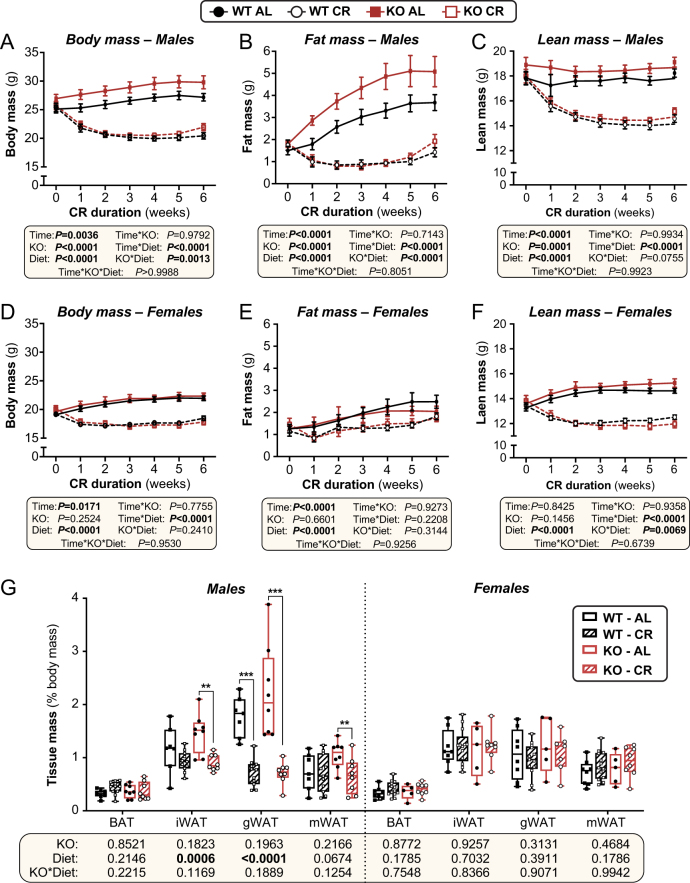
Effects of CR on body mass, composition, and adipose depot masses in WT and *Hsd11b1* KO mice. Male and female WT and *Hsd11b1* KO mice were fed *ad libitum* (AL) or a 30% CR diet from 9 to 15 weeks of age (0–6 weeks of CR). (A–F) Each week mice were weighed (A and D) and total fat mass (B and E) and lean mass (C and F) were measured by TD-NMR. (G) Masses of brown adipose tissue (BAT), inguinal WAT (iWAT), gonadal WAT (gWAT), and mesenteric WAT (mWAT) were recorded at necropsy and are shown as % body mass. Data are shown as mean ± s.e.m. (A–F) or as box-and-whisker plots (G) of the following numbers of mice per group: male WT AL, *n* = 7; male WT CR*, n* = 11; male KO AL*, n* = 8; male KO CR,*n* = 8; female WT AL, *n* = 8; female WT CR*, n* = 13; female KO AL*, n* = 5; female KO CR*, n* = 8. For A–F, significant effects of diet, sex, or time, and interactions thereof, were determined by mixed-effects models. For G, significant effects of diet and/or KO within each tissue were determined by two-way ANOVA with Šidák’s multiple comparisons tests. *P* values from ANOVA or mixed models are shown beneath the graphs, as indicated. For G, significant differences between comparable groups are indicated by **P < 0.01 or ***P < 0.001. Source data are provided as a Source Data file. See also Supplementary Fig. 4.

In males, CR decreased total body mass, fat mass, and lean mass in both genotypes (Fig. 2A, B, andC). Body mass and fat mass were greater in KO vs WT males on an AL diet, but not during CR; thus, the effects of CR on body mass and fat mass were stronger in KO than WT males (Fig. 2A and B: KO × diet *P* = 0.0005 or <0.0001). In females, CR decreased total body mass and lean mass but, consistent with our previous findings (Suchacki *et al.* 2023), CR-induced fat loss was far weaker than in males (Fig. 2D, E, and F). Unlike in males, in females *Hsd11b1* KO did not influence CR-induced weight loss or fat loss (Fig. 2D and E); however, CR decreased lean mass to a greater extent in KO vs WT females (Fig. 2F: KO × diet *P* = 0.0069).

We further investigated these effects at a tissue-specific level after 6 weeks of AL or CR diet. Neither CR nor KO affected brown adipose tissue (BAT) mass in males or females, whether for absolute mass (Supplementary Fig. 6A) or as % of body mass (Fig. 2G). In contrast, CR decreased the masses of iWAT, gWAT, and mWAT in males but not in females (Supplementary Fig. 6A and Fig. 2G). *Hsd11b1* KO alone did not affect the masses of any of these adipose depots, nor did it alter CR’s effects on these tissues (as evidenced by no significant KO × diet interactions). In females, *Hsd11b1* KO enhanced CR-induced decreases in kidney and spleen masses (Supplementary Fig. 6B, C, D, and E); however, KO alone did not affect the masses of the liver, kidneys, spleen, pancreas, or heart in males or females, and did not influence CR’s effect on the masses of these tissues in male mice (Supplementary Fig. 6B, C, D, and E).

### Hsd11b1 KO males resist CR-induced increases in circulating and BM corticosterone

**Figure 3:**
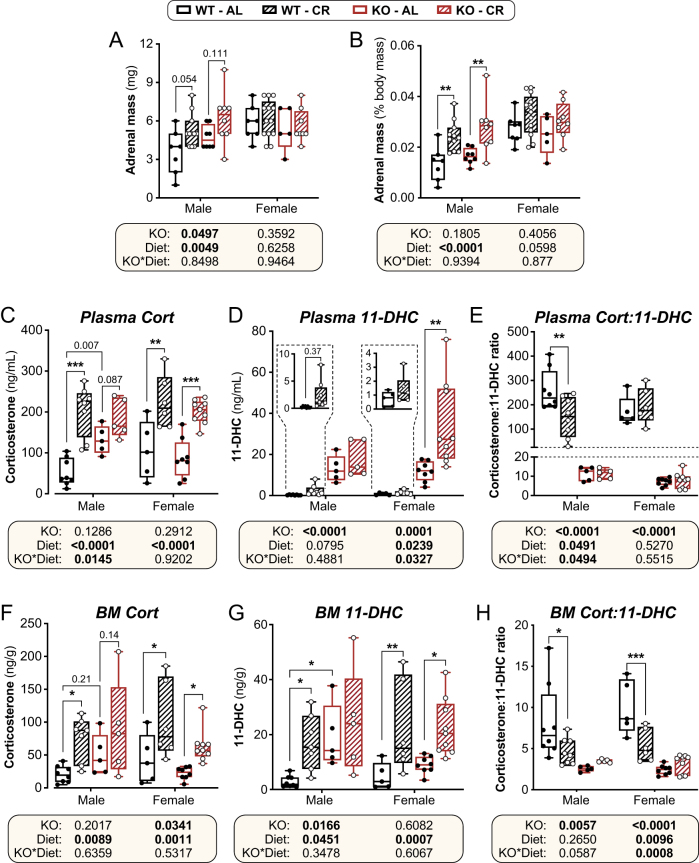
Effects of CR and *Hsd11b1* KO on adrenal mass and concentrations of corticosterone and 11-DHC in plasma and BM. Male and female WT and *Hsd11b1* KO mice were fed AL or a 30% CR diet as described in Fig. 2. (A and B) Adrenal glands from 15-week-old mice were weighed at necropsy. Masses are shown in grams (A) or as % body mass (B). (C–H) Tail vein blood and femoral BM were collected from 10-week-old mice at necropsy. Concentrations of corticosterone and 11-DHC in plasma (C and D) and BM (F and G) were then measured by LC-MS/MS and used to calculate the ratio of corticosterone: 11-DHC (E and H). Data are shown as box-and-whisker plots of the following numbers of mice per group: male WT AL, *n* = 7 (A and B) or 8 (C–H); male WT CR, *n* = 11 (A and B) or 11 (C–H); male KO AL*, n* = 8 (A and B) or 5 (C–H); male KO CR*, n* = 8 (A and B) or 5 (C–H); female WT AL, *n* = 8 (A and B) or 5 (C–H); female WT CR, *n* = 13 (A,B) or 6 (C–H); female KO AL*, n* = 5 (A and B) or 8 (C–H); female KO CR,*n* = 7 (A and B) or 9 (C–H). Significant effects of diet and/or KO within each sex were determined by two-way ANOVA with Šidák’s multiple comparisons tests (A and B) or Fisher’s LSD test (C–H). Overall ANOVA *P* values are shown beneath the graphs, while significant diet effects within each sex and genotype are indicated by *P < 0.05, **P < 0.01, or ***P < 0.001. Source data are provided as a Source Data file.

We next assessed the effects of CR and *Hsd11b1* KO on the HPA axis and glucocorticoid exposure, both systemically and within the BM. In males, 6 weeks of CR increased adrenal mass in WT and KO mice, particularly when expressed relative to body mass (Fig. 3A and B). KO males also had larger adrenals than WT males irrespective of diet (Fig. 3A; KO *P* = 0.0497). Both absolute and relative adrenal masses were greater in females than males, and unlike in males, absolute adrenal mass in females was unaffected by diet or genotype (Fig. 3A). However, relative to body mass, the adrenal mass of WT and KO females did tend to increase during CR (Fig. 3B; diet *P* = 0.0598), suggesting that it is relatively maintained in the face of decreasing body mass.

Despite the variable effects on adrenal mass, 1 week of CR increased plasma corticosterone in WT males, WT females, and KO females (Fig. 3C). There was no significant CR-induced increase in *Hsd11b1* KO males, largely because AL-fed *Hsd11b1* KO males had higher corticosterone than their WT littermates (Fig. 3C). *Hsd11b1* KO did not influence CR-induced hypercorticosteronaemia in females, and during CR, plasma corticosterone was similar between *Hsd11b1* KO and WT males and females (Fig. 3C). These CR effects were less apparent in our 6-week CR group because, for this group, AL mice have fasted before necropsy, which increased corticosterone and blunted detection of the CR effect (data not shown). Thus, we focused on the effects of 1-week CR. Corticosterone concentrations within the BM showed a similar pattern to those in plasma, being significantly increased by CR in WT males, WT females, and KO females, but not in KO males (Fig. 3F). These data show that CR increases corticosterone concentrations in the plasma and BM of WT males, and in females of both genotypes; however, this is attenuated in the *Hsd11b1* KO males, largely because they have elevated corticosterone concentrations on an AL diet.

To further establish the consequences of *Hsd11b1* KO, we measured 11-DHC concentrations in these plasma and BM samples. As shown in Fig. 3D, plasma 11-DHC was significantly higher in KO vs WT males and females, irrespective of diet. One week of CR also increased plasma 11-DHC in females, and this effect was stronger for KO mice (Fig. 3D). In the BM, 11-DHC concentrations were also higher in KO vs WT males, but not females, and were increased by CR in all groups except KO males (Fig. 3G). Importantly, in both plasma and the BM, the ratio of corticosterone:11-DHC was markedly lower in KO vs WT males and females, both on AL and CR diets (Fig. 3E and H). This is similar to the decreased plasma corticosterone:11-DHC ratio of *Hsd11b1* KO mice in the context of systemic inflammation (Verma *et al.* 2018) and is consistent with *Hsd11b1* KO preventing the conversion of 11-DHC to active corticosterone, both systemically and within the BM.

### Hsd11b1 deletion attenuates CR-induced BMAT expansion in male but not female mice

**Figure 4:**
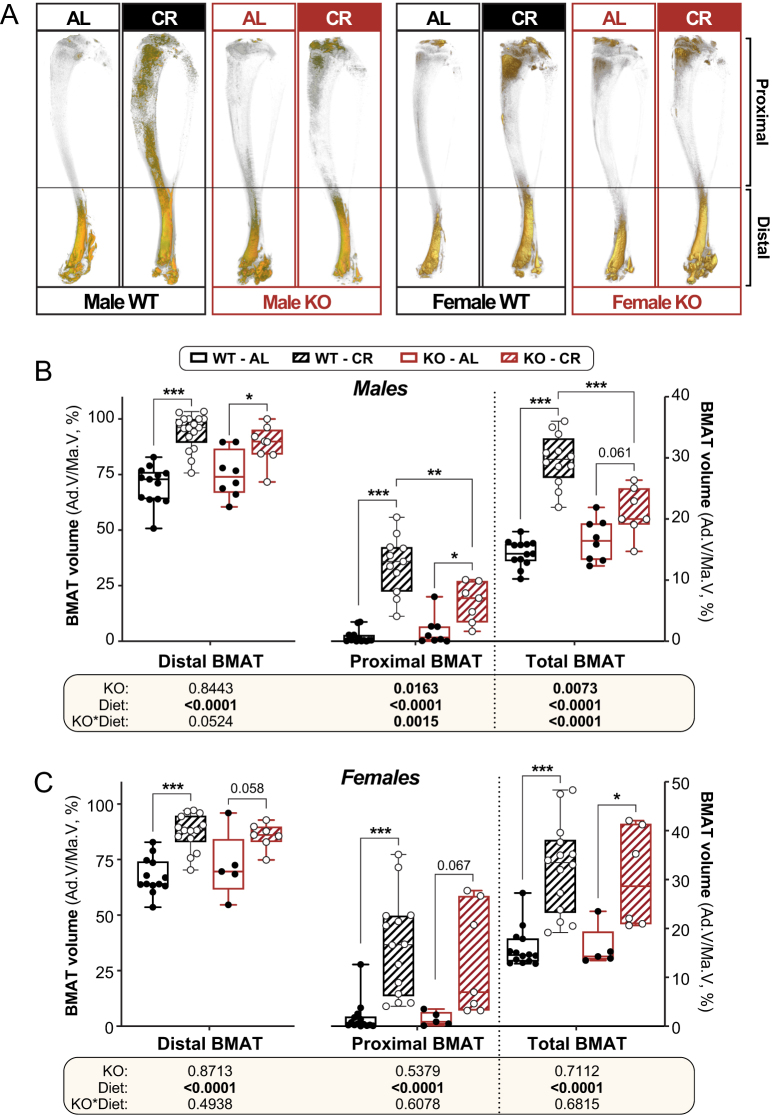
*Hsd11b1* KO attenuates CR-induced BMAT expansion in male but not female mice. Male and female WT and *Hsd11b1* KO mice were fed AL or a 30% CR diet as described for Figure 2. After necropsy, tibiae were stained with osmium tetroxide prior to µCT for analysis of BM adiposity. (A) Representative µCT scans of osmium tetroxide-stained bones. Stained regions of BMAT are shown in yellow. (B and C) BMAT volumes (Ad.V) from µCT scans of tibiae from males (B) and females (C), presented as % of marrow volume (Ma.V) for the distal, proximal, and total tibia. Data in (B and C) are box-and-whisker plots of the following numbers of mice per group: male *WT AL*, *n* = 13; male WT CR, *n* = 11; male KO AL, *n* = 8; male KO CR, *n* = 7; female WT AL, *n* = 13; female WT* CR*, *n* = 14; female KO AL, *n* = 5; female KO CR, *n* = 7. For (B), significant effects of diet and/or KO within each sex were determined by two-way ANOVA with Tukey’s multiple comparisons test. Overall ANOVA *P* values are shown beneath the graphs, while significance for multiple comparisons is shown as for Fig. 3. Source data are provided as a Source Data file.

We next investigated if *Hsd11b1* KO influenced CR-induced BMAT expansion, which is the critical test of our hypothesis. As shown in Fig. 4, 6 weeks of CR significantly increased BMAT in the proximal and distal tibia of WT males and females, with corresponding increases in total BMAT volume (Fig. 4A, B, and C). In males, *Hsd11b1* KO attenuated the CR-induced increases in proximal and total BMAT, with a significant KO × diet interaction occurring for each site (Fig. 4A and B). Indeed, among CR-fed mice, *Hsd11b1* KO males had significantly less proximal and total BMAT than their WT counterparts (Fig. 4A and B). KO also tended to blunt the increases in distal BMAT in males only (Fig. 4A: KO × diet *P* = 0.0524). In contrast, in females, *Hsd11b1* KO did not affect BMAT volume or its increase in response to CR (Fig. 4C). Thus, deletion of *Hsd11b1* attenuates CR-induced BMAT expansion in males but not females.

### Hsd11b1 deletion does not influence the effects of CR on trabecular or cortical bone

**Figure 5:**
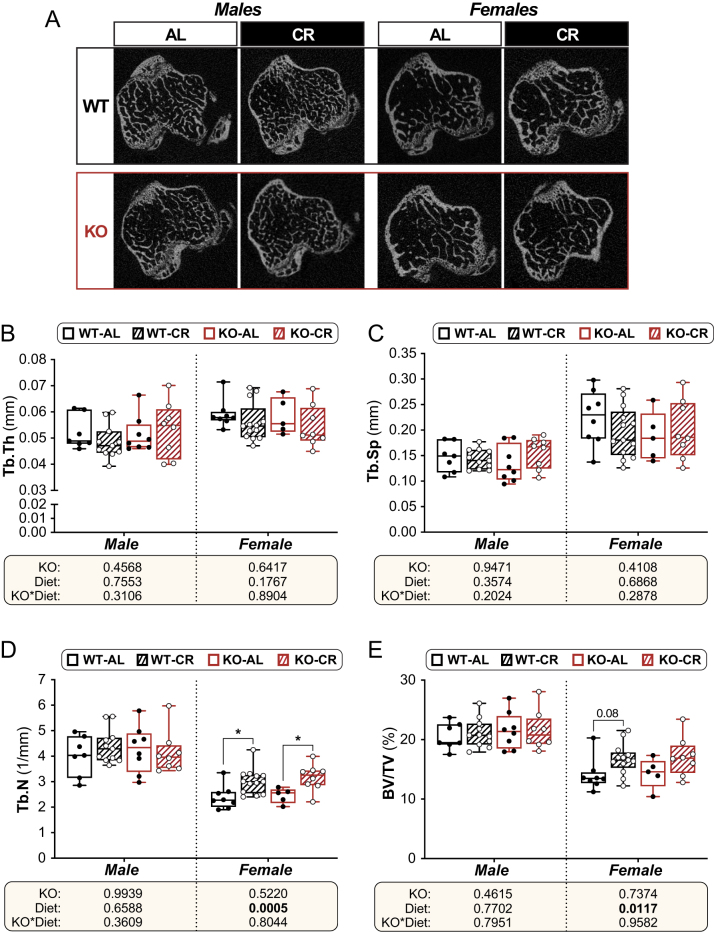
Effects of CR and *Hsd11b1* KO on trabecular bone in the proximal tibia. Male and female WT and *Hsd11b1* KO mice were fed AL or a 30% CR diet as described in Fig. 2. After necropsy, calcified tibiae underwent µCT for analysis of trabecular architecture. (A) Representative µCT images showing 2D axial sections of the proximal tibial metaphysis. (B) Trabecular thickness (Tb.Th), mm. (C) Trabecular separation (Tb.Sp), mm. (D) Trabecular number (Tb.N) per mm. (E) Trabecular bone volume fraction (BV/TV), %. Data in B–E are box-and-whisker plots of the following numbers of mice per group: male WT AL*, n* = 7; male WT CR*, n* = 11; male KO AL*, n* = 8; male KO CR*, n* = 8; female WT AL,*n* = 8; female WT CR,*n* = 13; female KO AL,*n* = 5; female KO CR*, n* = 8. For B–E, statistical analyses and presentation were done as described in Fig. 3. Source data are provided as a Source Data file.

**Figure 6:**
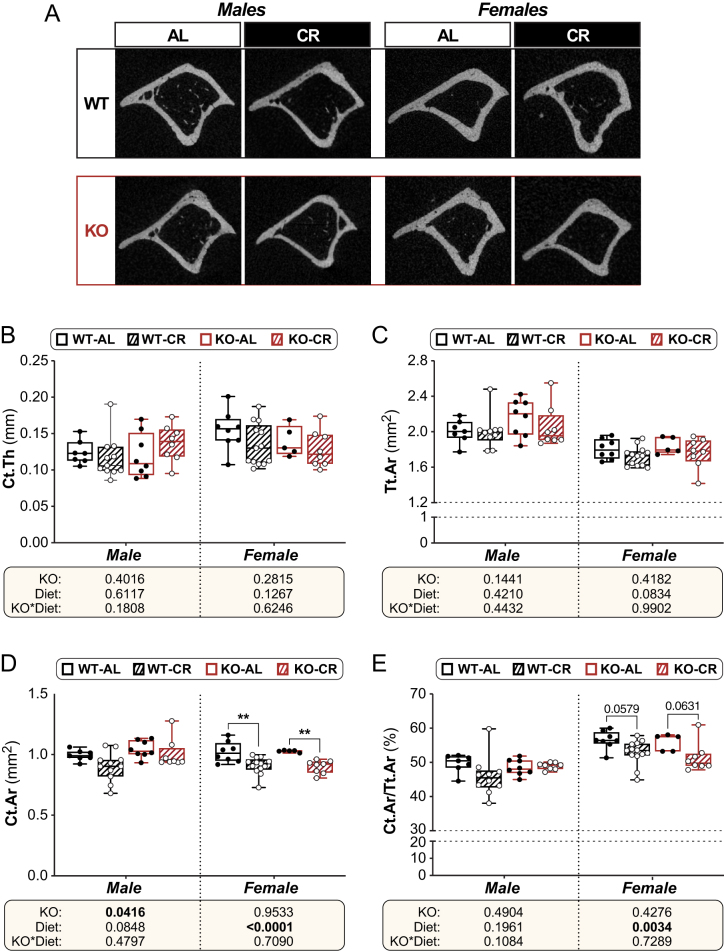
Effects of CR and *Hsd11b1* KO on cortical bone in the tibial diaphysis. Male and female WT and *Hsd11b1* KO mice were fed AL or a 30% CR diet as described in Fig. 2. After necropsy, calcified tibiae underwent µCT for analysis of cortical architecture in the proximal tibial diaphysis. (A) Representative µCT images showing 2D axial sections of the proximal tibial diaphysis. (B) Cortical thickness (Ct.Th), mm. (C) Total cross-sectional area inside the periosteal envelope (Tt.Ar), mm^2^. (D) Cortical bone area (Ct.Ar), mm^2^. (E) Cortical area fraction (Ct.Ar/Tt.Ar), %. For B–E, statistical analyses and presentation were done as described in Fig. 3. Source data are provided as a Source Data file.

BMAT expansion often coincides with bone loss, and 11β-HSD1 is expressed in bone and has been reported to influence skeletal remodelling (Fenton *et al.* 2019). Thus, we next investigated how CR and *Hsd11b1* KO affected trabecular and cortical architecture. As shown in Figs 5 and 6, in 15-week-old mice, after 6 weeks of CR or AL feeding, the effects of diet and genotype on trabecular and cortical bone were less pronounced than their effects on BMAT. Trabecular thickness and trabecular separation in the proximal tibia were unaffected by CR or *Hsd11b1* KO in males or females (Fig. 5B and C). In contrast, trabecular number and bone volume fraction were increased by CR in females of both genotypes but were not affected by CR in males, nor by KO in either sex (Fig. 5D and E). CR in males also did not influence any cortical parameters, including average cortical thickness (Ct.Th; Fig. 6B), total cross-sectional area inside the periosteal envelope (Tt.Ar; Fig. 6C), or cortical area fraction (Ct.Ar/Tt.Ar; Fig. 6E), although it tended to decrease cortical bone area (Ct.Ar; Fig. 6D, *P* = 0.0898). In females, CR decreased Ct.Ar and cortical area fraction and tended to decrease Tt.Ar but did not affect Ct.Th (Fig. 6B, C, D, and E). *Hsd11b1* KO increased Ct.Ar in males, regardless of diet (Fig. 6D), but otherwise KO did not affect any other cortical parameters nor influence the CR response in males or females.

### Hsd11b1 KO does not prevent CR from increasing glucocorticoid target gene expression in BM or WAT

**Figure 7:**
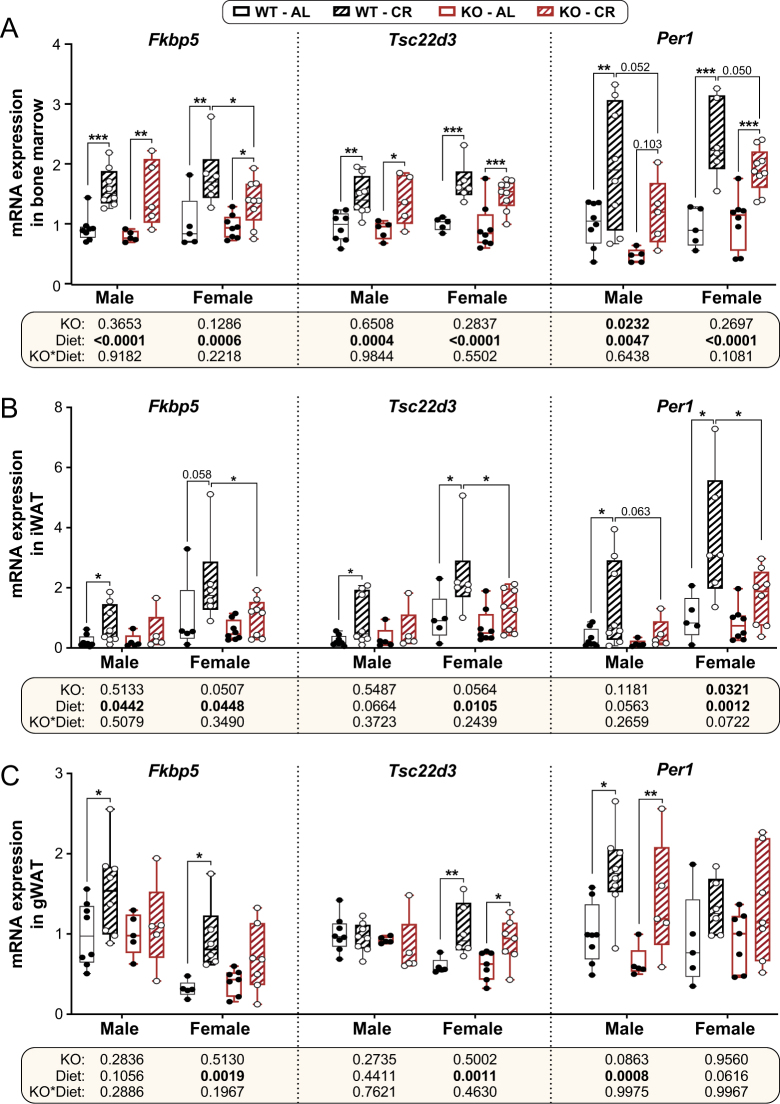
Effects of CR and *Hsd11b1* KO on mRNA expression of glucocorticoid target genes in BM and WAT. Male and female WT and *Hsd11b1* KO mice were fed AL or a 30% CR diet as described in Fig. 2. (A–C) Tibial BM, iWAT, and gWAT were collected from 10-week-old mice at necropsy and *Fkbp5, Tsc22d3,* and *Per1* mRNA levels were determined by qPCR. Expression of each mRNA is shown relative to levels in AL males or females after normalising to the geometric mean of the housekeeping genes *Ppia, Tbp,* and *Actb* (for BM) or *Ppia, Tbp,* and *Hprt* (for iWAT and gWAT). Box-and-whisker plots include the following numbers of mice per group: male WT AL, *n* = 8; male WT CR,*n* = 8; male KO AL,*n* = 5; male KO CR,*n* = 5; female WT AL, *n* = 5; *f*emale WT CR*, n* = 6; female KO AL,*n* = 8 (BM, iWAT) or 7 (gWAT); female KO CR*, n* = 9 (BM, iWAT) or 7 (gWAT). Within each tissue, significant effects of diet and/or KO within each sex were determined by two-way ANOVA. Overall ANOVA *P* values are shown beneath the graphs. Significant diet effects (within each sex and genotype) or genotype effects (within each sex and diet) were determined by Fisher’s LSD test and are indicated by *P < 0.05, **P < 0.01, or ***P < 0.001. Source data are provided as a Source Data file.

The above data show that, despite *Hsd11b1* KO having few effects on bone, male KO mice resist CR-induced increases in both BMAT and corticosterone concentrations in BM and plasma. Therefore, we tested if these effects are associated with altered glucocorticoid activity in BM or other tissues. To do so, we used qPCR to measure the expression of the glucocorticoid target genes *Fkbp5, Tsc22d3* (also known as *Gilz*), and *Per1* (D’Adamio *et al.* 1997, So *et al.* 2009, Lee *et al.* 2011, Reddy *et al.* 2012, Suarez *et al.* 2012, Pereira *et al.* 2014) in BM and WAT from the same mice that had undergone LC–MS measurements of corticosterone and 11-DHC (Fig. 3C, D, E, F, G and H). Within the BM, CR increased the expression of each of these target genes in males and females (Fig. 7A). Across both diets, *Hsd11b1* KO males had significantly lower BM *Per1* expression than their WT littermates (Fig. 7A; KO *P* = 0.0232 for male *Per 1*). However, in males and females, there were no other overall KO effects for any of the other transcripts in the BM (Fig. 7A). *Hsd11b1* KO mildly influenced the induction of these genes during CR, including significant attenuation of increased *Fkbp5* expression in females, and a trend toward lower induction of *Per1* during CR in both sexes. However, none of the transcripts showed significant KO × diet interactions, demonstrating that, unlike for male BMAT, *Hsd11b1* KO does not prevent CR from increasing glucocorticoid activity within the BM.

*Hsd11b1* KO mice resist the effects of exogenous glucocorticoid excess on WAT, including the induction of genes related to lipid metabolism (Morgan *et al.* 2014). Therefore, we also investigated if CR activates glucocorticoid activity in WAT, and if the KO mice resist this. In WT males and females, CR increased *Fkbp5*,*Tsc22d3*, and *Per1* expression in iWAT (Fig. 7B), and *Fkbp5* in gWAT (Fig. 7C). CR also increased gWAT expression of *Tsc22d3* in females, and *Per1* in males (Fig. 7C). These data are consistent with CR increasing glucocorticoid activity in iWAT and gWAT. We next assessed if *Hsd11b1* KO alters this effect. Across both diets, *Hsd11b1* KO significantly decreased *Per1* expression and tended to decrease *Fkbp5* and *Tsc22d3* in female iWAT (Fig. 7B). In contrast, in iWAT of males and gWAT of each sex, KO had no overall effect on the expression of these transcripts (Fig. 7B and C). Thus, irrespective of diet, *Hsd11b1* KO tends to decrease glucocorticoid-target gene expression in iWAT of females but not males. In terms of the CR response, KO significantly attenuated the induction of each gene in iWAT of females, suggesting that 11β-HSD1 is important for the full effect of CR on these glucocorticoid target genes. Although there was a trend for *Hsd11b1* KO to blunt induction of *Per1* in male iWAT, there was no effect on the induction of *Fkbp5* or *Tsc22d3* (Fig. 7B). This suggests that 11β-HSD1 contributes little to the effect of CR on glucocorticoid-target genes in male iWAT. The CR response in both male and female gWAT was also similar between WT and KO mice (Fig. 7B and C).

### Male KO mice have increased progesterone in plasma and BM

These qPCR data indicate that CR increases glucocorticoid activity in BM and iWAT and that 11β-HSD1 makes little contribution to this effect in male mice. This suggests that mechanisms other than glucocorticoid activity or intracellular glucocorticoid levels are responsible for the attenuation of CR-induced BMAT expansion in male *Hsd11b1* KO mice. Therefore, we next investigated this possibility.

**Figure 8:**
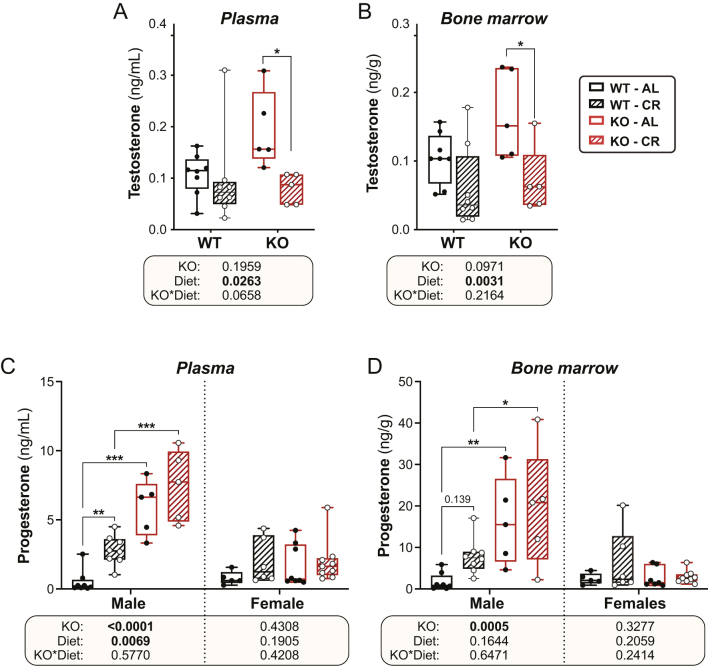
Effects of CR and *Hsd11b1* KO on testosterone and progesterone in plasma and BM. Male and female WT and *Hsd11b1* KO mice were fed AL or a 30% CR diet as described in Fig. 2. (A–D) Tail vein blood and femoral BM were collected from 10-week-old mice at necropsy. Concentrations of testosterone (A and B) and progesterone (C and D) were then measured by LC-MS/MS. Data are shown as box-and-whisker plots of the following numbers of mice per group: male WT AL, *n* = 8; male WT CR*, n* = 8; male KO AL*, n* = 5; male KO CR,*n* = 5; female WT AL, *n* = 5; female WT CR*, n* = 6; female KO AL*, n* = 8; female KO CR*, n* = 9. Statistical analyses and presentation are as described in Fig. 7. Source data are provided as a Source Data file.

Androgens can suppress BMAT formation (Tamura *et al.* 2005). In ageing men, BMAT expansion is associated with decreased circulating testosterone (Mistry *et al.* 2018). Moreover, CR decreases testosterone concentrations in healthy, lean men (Cangemi *et al.* 2010). Therefore, we measured testosterone concentrations in plasma and BM to investigate whether testosterone is associated with the differential effects of CR and KO on BMAT expansion. Testosterone was detectable in the BM of one WT CR female and one KO CR female, but not in the BM or plasma of the remaining female mice, regardless of diet or genotype (data not shown). In males, CR significantly decreased testosterone concentrations in both plasma and BM, and this was not influenced by *Hsd11b1* KO (Fig. 8A and B). Thus, the resistance of male *Hsd11b1* KO mice to CR-induced BMAT expansion is not associated with an alteration in testosterone during CR.

Another steroid hormone that inhibits BMAT accumulation is 17β-oestradiol (Cawthorn 2020). We recently reported that CR activates oestrogen-related signaling in male mice (Suchacki *et al.* 2023). Thus, we hypothesised that the KO males might resist CR-induced BMAT accumulation owing to increases in oestrogen action, and that BMAT expansion in females might be driven primarily by decreased oestradiol during CR. Unfortunately, accurate measurement of oestrogens by LC-MS/MS is not yet possible in the very low amounts of plasma and BM available from mice; hence, we were unable to quantify 17β-oestradiol in our samples. However, our LC-MS/MS method allowed analysis of other steroid hormones, including progesterone. This revealed a striking genotype effect in male mice, with plasma and BM progesterone concentrations being much higher in *Hsd11b1* KO males compared to their WT littermates (Fig. 8C and D). This was not apparent in females, where concentrations remained similar between *Hsd11b1* KO and WT mice. Moreover, in WT male mice, CR increased progesterone concentrations in plasma and tended to increase these in the BM, but this did not occur in *Hsd11b1* KO males or in females of either genotype (Fig. 8C and D). These observations highlight the possibility that increased progesterone action contributes to the resistance of *Hsd11b1* KO males to CR-induced BMAT expansion.

## Discussion

Our data robustly demonstrate that global ablation of *Hsd11b1* does not affect BM adiposity in adult mice fed a normal chow diet and instead suggests a role for 11β-HSD1 in CR-induced BMAT expansion in males but not in females. The ability of *Hsd11b1* KO to attenuate CR-induced increases in plasma and BM corticosterone concentrations, which occurs in male but not female mice, is consistent with this. However, *Hsd11b1* KO does not prevent CR from increasing glucocorticoid-target gene expression within the BM, suggesting that decreased glucocorticoid activity is not the mechanism. Given that glucocorticoids directly impact bone remodeling (Fenton *et al.* 2019), the lack of difference between WT and *Hsd11b1* KO mice in CR’s effects on trabecular or cortical bone further suggests mechanisms other than glucocorticoids. Unexpectedly, male (but not female) *Hsd11b1* KO mice were discovered to have elevated plasma and BM progesterone concentrations. The increase in progesterone concentrations in response to CR in WT mice, with no further increase in the already high levels in *Hsd11b1* KO mice, identifies progesterone as a potential modulator of CR-induced BMAT expansion that may also influence other effects of CR.

### Effects of CR on Hsd11b1 expression and glucocorticoid activity within the BM

We demonstrate, for the first time, that CR increases corticosterone concentrations within the BM and that this is associated with increased BM expression of glucocorticoid-target genes. For all four steroids analysed (corticosterone, 11-DHC, testosterone, and progesterone), the BM concentrations generally mirror those within the plasma, suggesting that plasma levels are indicative of those within the BM. Interestingly, recent RNAseq data from Liu *et al.,* who studied CR in male mice (Li *et al.* 2022*b*), also show increased BM expression of *Fkbp5*, *Tsc22d3*, and *Per1* following CR. Their study did not directly report these transcriptional effects or focus on glucocorticoid activity; however, their data further support our conclusion that CR robustly enhances glucocorticoid action within the BM.

The ability of CR to increase BM *Hsd11b1* expression – itself a glucocorticoid-target gene in some tissues (Tomlinson *et al.* 2004) – plausibly could contribute to elevated BM corticosterone during CR. However, this does not appear to be the case: although the increase in *Hsd11b1* mRNA is more robust in females than males, the increase in BM corticosterone is similar in both sexes. Furthermore, CR in WT mice decreases the corticosterone:11-DHC ratio, which suggests lower rather than higher 11β-HSD1 activity. This decreased ratio is unlikely to result from 11β-HSD2, because we confirm that this enzyme is expressed at very low levels within mouse BM (Thorrez *et al.* 2008) and show that this is not increased in CR. Instead, it may be that CR causes a high flux of 11-DHC from plasma to BM, saturating 11β-HSD1 and thereby decreasing the corticosterone:11-DHC ratio.

It is unclear why CR does not further increase plasma or BM corticosterone in *Hsd11b1* KO males. Even on an AL diet, these mice have larger adrenals and increased plasma and BM corticosterone than their WT counterparts, suggesting elevated HPA axis activity. Although elevated plasma corticosterone has been observed in male *Hsd11b1* KO mice on a mixed MF1 background (Carter *et al.* 2009), other studies of total *Hsd11b1* KO males on a C57BL/6 background find no increase in plasma corticosterone (Abrahams *et al.* 2012, Verma *et al.* 2018). Given that our mice are also on a C57BL/6 background, it could be that our AL males have increased corticosterone as a result of single housing, which can stimulate the HPA axis (Hebda-Bauer *et al.* 2019). If so, one possibility is that the elevated plasma and BM corticosterone represent a ceiling of adrenal output that cannot be further stimulated by CR. However, the underlying reasons are unknown and merit further investigation.

### Progesterone as a regulator of BMAT formation

Our finding that CR and *Hsd11b1* KO each increase progesterone concentrations, especially in male mice, is novel and raises the broader question of whether (and how) progesterone influences the effects of CR and *Hsd11b1* KO, including on BMAT expansion. There are several intriguing possibilities. Firstly, progesterone can inhibit 11β-HSD1’s reductase activity but stimulate its dehydrogenase activity (Tomlinson *et al.* 2004). Thus, increased progesterone during CR may serve to limit excessive glucocorticoid exposure within the BM and other tissues, at least when 11β-HSD1 is present.

Secondly, progesterone might influence BMAT expansion via steroid hormone receptors. Microarray data confirm that the progesterone receptor (PR) is expressed in both BM (Thorrez *et al.* 2008) and BMAT (Suchacki *et al.* 2020), and progesterone can also bind directly to the glucocorticoid receptor (GR) to modulate its activity. In some cases, progesterone competes with glucocorticoids for GR binding, thereby suppressing GR activity in a concentration-dependent manner (Ganguly *et al.* 1982). In other contexts, progesterone promotes GR-dependent transcriptional activation (Lei *et al.* 2012). A further complexity is that the PR and GR can also form heterocomplexes that suppress or augment the effects of their respective hormones (Pecci *et al.* 2022). Consequently, progesterone and glucocorticoids synergistically activate the expression of some genes but antagonise each other’s effects on other transcriptional targets (Pecci *et al.* 2022). Thus, during CR in WT mice, increased progesterone may be contributing to BMAT expansion by stimulating GR activity, whereas the even-greater progesterone concentrations in *Hsd11b1* KO males might suppress the activation of other GR targets and thereby attenuate BMAT expansion.

One caveat to the above interpretations is our finding that *Hsd11b1* KO generally does not affect glucocorticoid activity within the BM, at least based on the GR target genes assessed. This suggests that other mechanisms are involved. Notably, progesterone can also modulate the response to oestrogens (Pecci *et al.* 2022), which are potent inhibitors of BMAT expansion (Cawthorn 2020). Therefore, a third possibility is that, at the very high concentrations occurring in *Hsd11b1* KO males, progesterone attenuates BMAT expansion by exerting oestrogen-like effects.

A separate possibility relates to the effects of sex hormones, CR, and *Hsd11b1* KO on body temperature. CR in mice decreases core body temperature and this is prevented by progesterone or oestradiol administration, at least in ovariectomised females (Cintron-Colon *et al.* 2019). Given that *Hsd11b1* ^hypo^ males have increased core body temperature (Morton *et al.* 2004), it may be that total *Hsd11b1* KO alters the hypothermic effects of CR and thereby influences sex steroid production to compensate for dysregulation of core temperature.

Despite these possibilities, it remains unclear why progesterone concentrations increase during CR in WT mice and are elevated even further in *Hsd11b1* KO males. Progesterone is the most abundant sex hormone in orchidectomised mice (Colldén *et al.* 2022), suggesting that the effects of CR and *Hsd11b1* KO in males could relate to changes in gonadal function. This, and the above possibilities regarding progesterone as a regulator of BMAT expansion, are important issues meriting further investigation.

### Roles of progesterone in other effects of CR

Progesterone also exerts many metabolic effects, including suppressing gluconeogenesis and stimulating glucose uptake (Kalkhoff 1982, Zhang *et al.* 2020); promoting lipid storage and inhibiting lipolysis in WAT (Stelmanska *et al.* 2015); and acting with 17β-oestradiol to increase fasting ketones (Kalkhoff 1982). Several of these effects are relevant to sex differences in the CR response, including decreased lipolysis and greater ketogenesis in females than in males (Suchacki *et al.* 2023). Progesterone can also target many molecular pathways regulated by CR (Lee *et al.* 2012, Choi *et al.* 2014, Fedotcheva *et al.* 2022); however, whether progesterone influences the CR response remains unknown. There is growing interest in how sex differences influence health and disease. Therefore, the role of progesterone during CR, including on metabolic and skeletal function, is an intriguing question for future research.

### CR, bone loss, and BMAT expansion

Our results also shed new light on the skeletal effects of CR, including sex differences and the interplay between bone loss and BMAT. We show that CR’s effects on cortical and trabecular bone are stronger in females than in males and vary depending on the skeletal site: while CR decreases cortical bone area and cortical area fraction, it has no effect on any trabecular parameter in males and actually increases trabecular number and bone volume fraction in females. The latter is consistent with numerous other mouse studies showing that CR decreases cortical bone while trabecular bone is either maintained or increased (Hamrick *et al.* 2008, Cawthorn *et al.* 2014, Mitchell *et al.* 2015, Devlin *et al.* 2016, Maridas *et al.* 2019, Pierce *et al.* 2019). Devlin *et al* found that CR caused robust trabecular bone loss, but their mice began CR when only 3 weeks old (Devlin *et al.* 2010). Thus, the timing and extent of CR likely influence its skeletal effects.

Mouse housing conditions may also impact the CR response. Most mouse CR studies, including our present research, house mice individually at room temperature. Compared to housing at thermoneutrality, mice housed at room temperature (~22ºC) have trabecular bone loss and lower BM adiposity (Iwaniec *et al.* 2016). The latter is prevented by beta-adrenergic inhibition, suggesting that room temperature housing decreases BMAT, in part, by increasing sympathetic nervous system activity (Turner *et al.* 2020). This is notable because CR may suppress sympathetic activity (Niemann *et al.* 2021), which could therefore be another mechanism contributing to CR-induced BMAT expansion; however, it is unclear if housing temperature would confound any effect of *Hsd11b1* KO on the CR response. Thus, future studies should investigate if thermoneutral housing influences CR’s effects on BMAT and the HPA axis, as tested recently for other effects of CR (Guijas *et al.* 2020).

Despite these additional considerations, an important finding of the present study is that CR maintains or increases trabecular bone volume despite robustly increasing BMAT. This is compelling evidence that BMAT expansion is not sufficient to drive bone loss. Indeed, a recent paper shows that ablation of BMAT exacerbates bone loss in CR (Li *et al.* 2022*b*). Thus, rather than promoting bone loss, BMAT may serve to support bone maintenance during systemic energy deficit.

### Hsd11b1 KO as a tool to restrain glucocorticoid activity: limitations and alternative approaches

The fact that *Hsd11b1* KO does not prevent CR-induced increases in glucocorticoid target genes highlights the need for other experimental approaches to test if glucocorticoids contribute to BMAT expansion during CR. Adrenalectomy robustly blocks HPA activity but is challenging during CR because adrenalectomised mice struggle to adapt to food deprivation, leading to increased mortality (Pashko & Schwartz 1992). Instead, tissue-specific deletion of the GR may be a more tractable approach. A particularly notable study is from Pierce *et al,* who used Osx1-Cre to delete GR in osteoprogenitors and investigated if this altered CR’s effects on bone and BMAT (Pierce *et al.* 2019). They found that GR deletion does not alter bone loss or BMAT expansion in response to CR, suggesting that these CR effects are independent of glucocorticoid activity. Importantly, they studied female mice only; hence, their findings are consistent with our observation that *Hsd11b1* KO does not influence CR-induced BMAT expansion in females. While it would be informative to extend their study to male mice, one caveat is that, using Osx1-Cre, GR deletion alone causes bone loss and increased tibial BMAT (Pierce *et al.* 2019). This complicates interpretation of the CR response. An alternative approach would be GR deletion using the recently developed BM adipocyte-specific Cre mice (Li *et al.* 2022*b*). This novel model opens the possibility of testing if glucocorticoid action, in BM adipocytes alone, contributes to CR’s skeletal effects.

In summary, our results reveal new knowledge about how CR influences glucocorticoid activity; the impact of CR and 11β-HSD1 deficiency on adiposity and bone; the mechanisms of CR-induced BMAT expansion; the relationship between BMAT and bone loss; and sex differences in these diverse phenomena. The finding that CR increases progesterone in WT male mice is particularly intriguing and warrants further studies to determine the roles of progesterone in the CR response.

## Supplementary Materials

Supplementary Figures

## Declaration of interest

The authors declare that there are no conflicts of interest that could be perceived as prejudicing the impartiality of the research reported herein. KEC is a Senior Editor of the *Journal of Endocrinology* and the *Journal of Molecular Endocrinology*. KEC was not involved in the review or editorial process for this paper, on which she is listed as an author.

## Funding

This work was supported by grants from the Medical Research Councilhttp://dx.doi.org/10.13039/501100000265 (MR/ M021394/1 to WPC, including support for KJS and BJT), the University of Edinburgh (Chancellor’s Fellowship to WPC; PhD Studentship to AL), the British Heart Foundationhttp://dx.doi.org/10.13039/501100000274 (BHF) (4-year BHF PhD Studentship to BJT and RJS), and the Welcome Trust (NIA grant 100981/Z/13/Z to NMM).

## Rights retention statement

For the purpose of open access, the author has applied a Creative Commons Attribution (CC-BY) licence to any Author Accepted Manuscript version arising from this submission.

## Author contribution statement

Contributions are based on the CRediT (Contributor Roles Taxonomy) and are as follows: conceptualisation, AL, KEC, and WPC; data curation, AL, KJS, BJT, NZMH, and WPC; formal analysis, AL, KJS, FR, NZMH, and WPC; funding acquisition, NZMH, KEC, and WPC; investigation, AL, KJS, FR, RJS, RJW, BJT, RMBB, IPC, GM, NZMH, and WPC; methodology, AL, KJS, RJW, GM, NZMH, KEC, and WPC; project administration, NZMH, KEC, and WPC; resources, NMM, NZMH, KEC, and WPC; supervision, NZMH, KEC, and WPC; visualisation, AL, KJS, KEC, and WPC; writing – original draft, AL and WPC; writing – review and editing, NZMH, KEC, and WPC.

## Acknowledgements

LC-MS/MS data were obtained at the University of Edinburgh, Mass Spectrometry Core, RRID:SCR_021833 with data collected on the SCIEX QTRAP 6500+ instrument (RRID:SCR_021831). We thank Tricia Lee and Scott Denham of the Mass Spectrometry Core for their technical assistance and acknowledge the financial support of NHS Research Scotland (NRS) for the Mass Spectrometry Core, Edinburgh Clinical Research Facility. Finally, we thank all staff at Edinburgh Bioresearch & Veterinary Services for their superb technical support.
