# Supplementary material for: Deletion of Hsd11b1 suppresses caloric restriction-induced bone marrow adiposity in male but not female mice
Source: J Endocrinol. 2024 Jun 24;262(2):e240072. doi: 10.1530/JOE-24-0072 (PMC11301425; doi:10.1530/JOE-24-0072)
Supplement: Supplementary Figures [file supplementary_figures.pdf]

Supplementary Figure 1

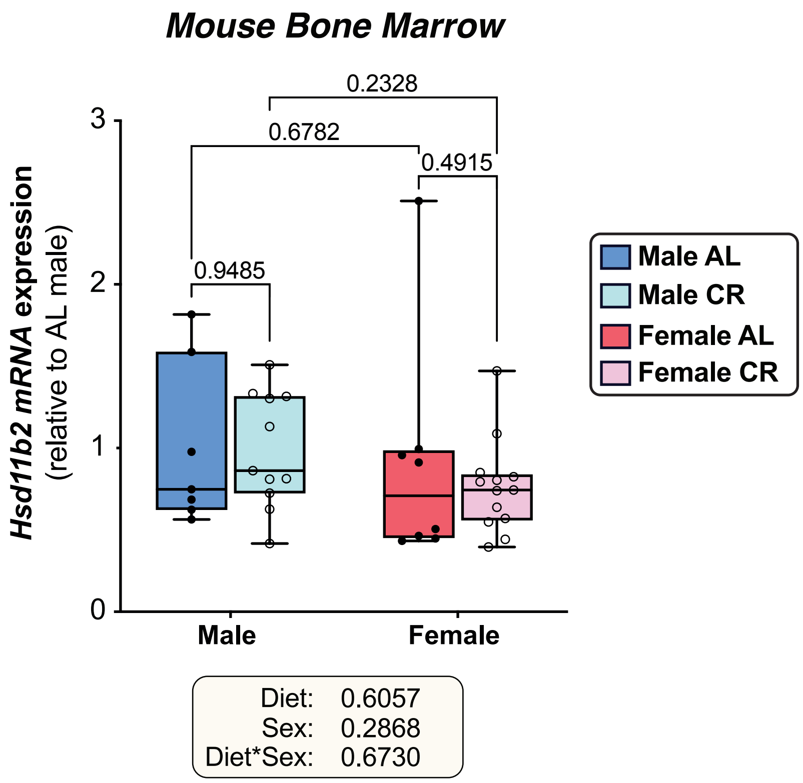

Supplementary Figure 1 – Transcripts encoding 11β-HSD2 are expressed at low levels within the BM and this is unaffected by six weeks of CR.
(A) Male and female mice on a C57BL/6JOlaHsd background were fed *ad libitum* (AL) or a 30% CR diet from 9-15 weeks of age (0-6 weeks of CR). At necropsy (15 weeks' old) tibial BM was sampled and expression of *Hsd11b2* was determined by qPCR. Expression is shown relative to levels in AL males after normalising to the geometric mean of the housekeeping genes *Ppia, Tbp* and *Actb*. Data expression, numbers per group, and statistical analyses are as described for Figure 1A. Source data are provided as a Source Data file.

Supplementary Figure 2

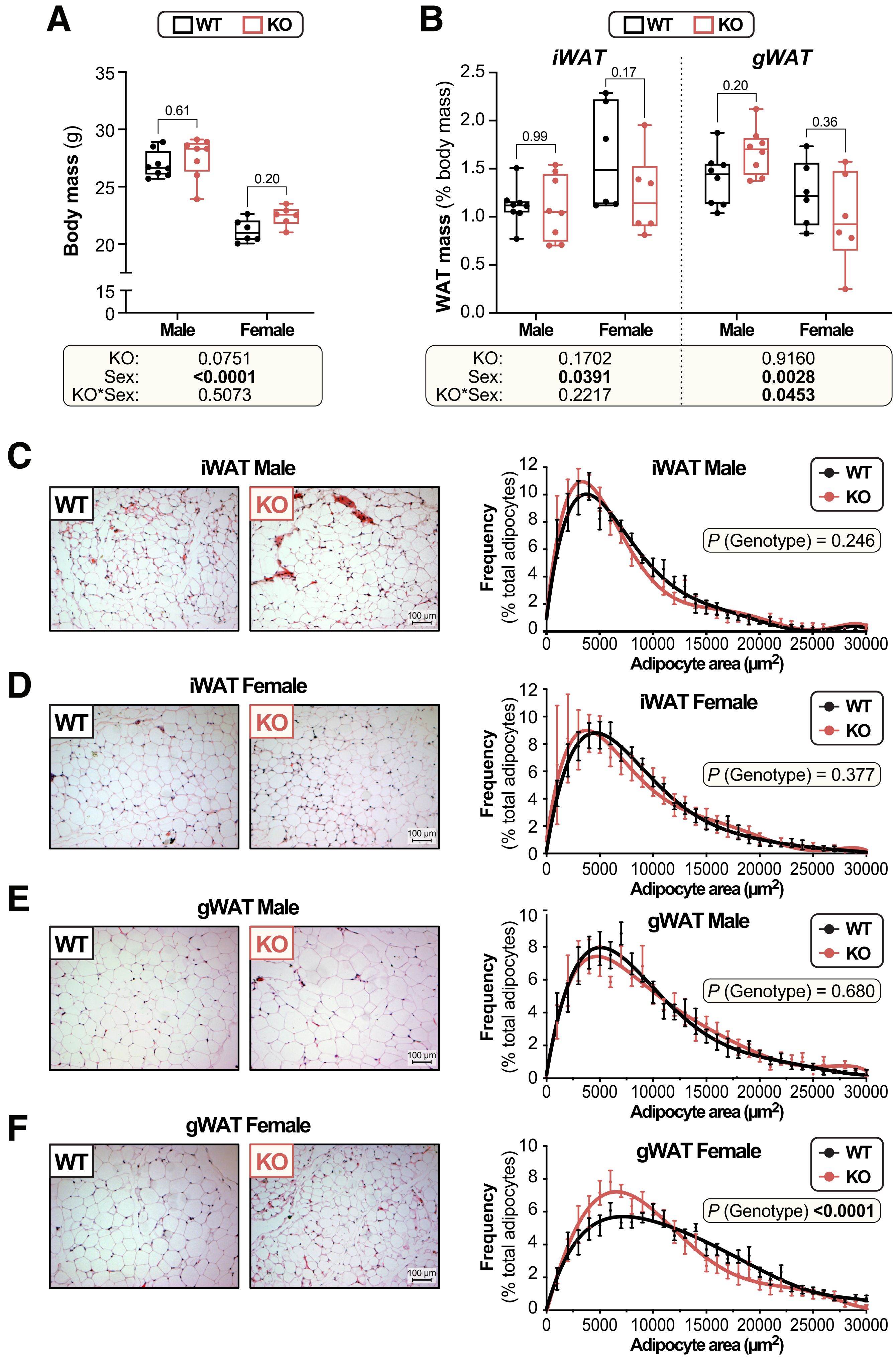

Supplementary Figure 2 – Global deletion of *Hsd11b1* does not affect BM adiposity in young adult mice.

Male and female WT and *Hsd11b1* KO mice on a C57BL/6JOlaHsd background were maintained on a chow diet until 13 weeks of age. At necropsy (13 weeks' old) body masses were recorded **(A)** and iWAT and gWAT were sampled and weighed **(B)**. Box-and-whisker plots in (A-B) include the following numbers of mice per group: *male WT,* n=8; *female WT,* n=6; *male KO,* n=8; *female KO,* n=6. Significant effects of *Hsd11b1* KO, sex, and KO*sex interactions were determined by 2-way ANOVA, with *P values* shown beneath each graph. For each measurement (body mass, iWAT mass, or gWAT mass), significant effects of KO were determined by Šídák's multiple comparisons test; *P* values for each pairwise comparison are shown on the graphs. **(C-F)** Adipocyte sizes were quantified by histomorphometry. Representative micrographs of H&E-stained sections are shown on the left of each panel (scale bar, 100 µm). Corresponding graphs of adipocyte size distribution are shown on the right of each, with the frequency of adipocytes within each size range presented as mean ± SEM. Data in (C-F) represent the following number of mice: iWAT = 8 male WT, 8 male KO, 6 female WT, 6 female KO; gWAT = 6 male WT, 7 male KO, 7 female WT, 6 female KO. For each sex and tissue, significant effects of genotype were determined by 2-way ANOVA. *P* values are shown on each graph.

Supplementary Figure 3

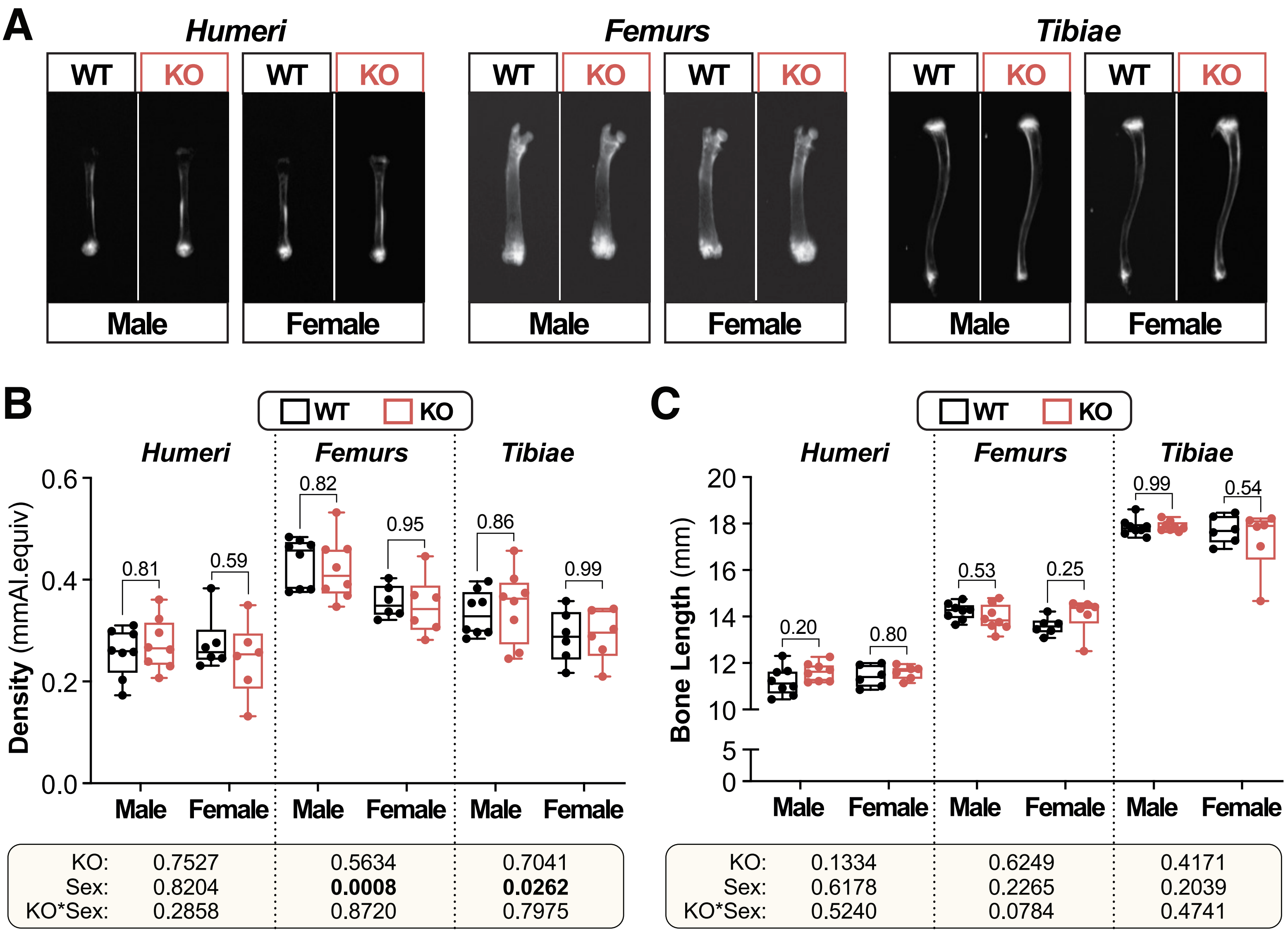

Supplementary Figure 3 – Global deletion of *Hsd11b1* does not affect bone density or length in young adult mice

Male and female WT and *Hsd11b1* KO mice were maintained on a chow diet until 13 weeks of age, as described for Supplementary Figure 1. At necropsy (13 weeks' old) long bones were dissected, fixed in formalin and analysed by X-ray. **(A)** Representative X-ray images of humeri, femurs and tibiae. **(B-C)** The density (B) and length (C) of bones, as determined from X-ray images. Data are shown as box-and-whisker plots of the following numbers of mice per group: *male WT,* n=8; *female WT,* n=6; *male KO,* n=8; *female KO,* n=6. Significant effects of *Hsd11b1* KO, sex, and KO*sex interactions were determined by 2-way ANOVA, with *P values* shown beneath each graph. Within each sex, significant effects of KO on the density or length of each bone type were determined by Šídák's multiple comparisons test; *P* values for each pairwise comparison are shown on the graphs.

Supplementary Figure 4

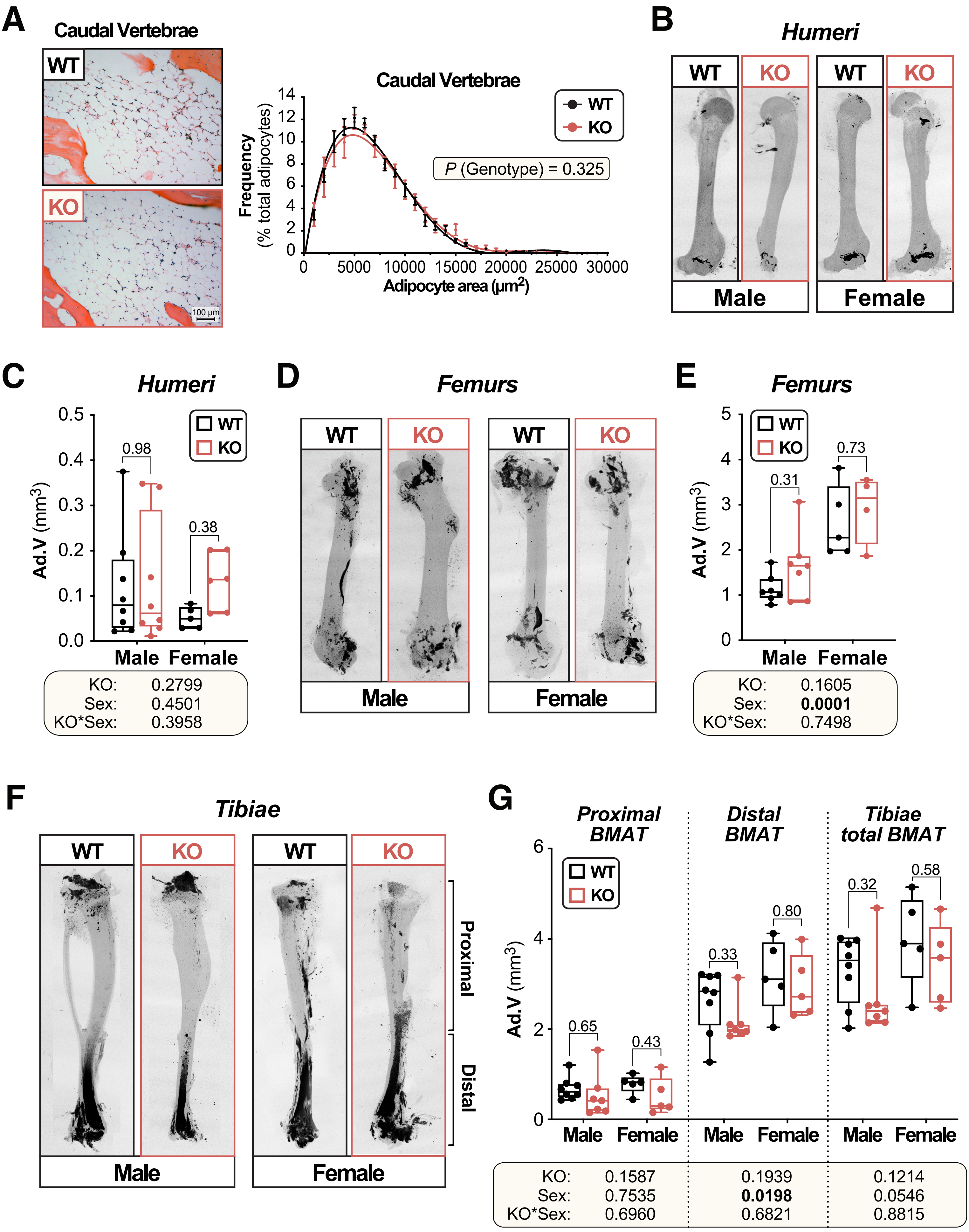

Supplementary Figure 4 – Global deletion of *Hsd11b1* does not affect bone marrow adiposity in young adult mice

Male and female WT and *Hsd11b1* KO mice were maintained on a chow diet until 13 weeks of age, as described for Supplementary Figure 1. At necropsy (13 weeks' old) long bones and caudal vertebrae were collected for analysis of BM adiposity. **(A)** Adipocyte sizes in caudal vertebrae of male mice were quantified by histomorphometry. Representative micrographs of H&E-stained sections (scale bar, 100 µm) and corresponding graphs of adipocyte size distribution are shown; for the latter, the frequency of adipocytes within each size range are presented as mean ± SEM. **(B-G)** Long bones were stained with osmium tetroxide prior to µCT for analysis of BM adiposity. Representative µCT scans are shown in (B), (D) and (F). The corresponding quantification of BMAT volumes (Ad.V) are shown in (C), (E) and (G) as box-and-whisker plots of the following numbers of mice per group: *male WT,* n=8 (humeri, tibiae) or 7 (femurs); *female WT,* n=5; *male KO,* n=8 (humeri) or 7(femurs, tibiae); *female KO,* n=6 (humeri), 5 (tibiae) or 4 (femurs). Ad.V for tibiae is shown for proximal, distal and total BMAT, as indicated. effects of *Hsd11b1* KO, sex, and KO*sex interactions were determined by 2-way ANOVA, with *P values* shown beneath each graph. Within each sex, significant effects of KO on the density or length of each bone type were determined by Šídák's multiple comparisons test; *P* values for each pairwise comparison are shown on the graphs.

Supplementary Figure 5

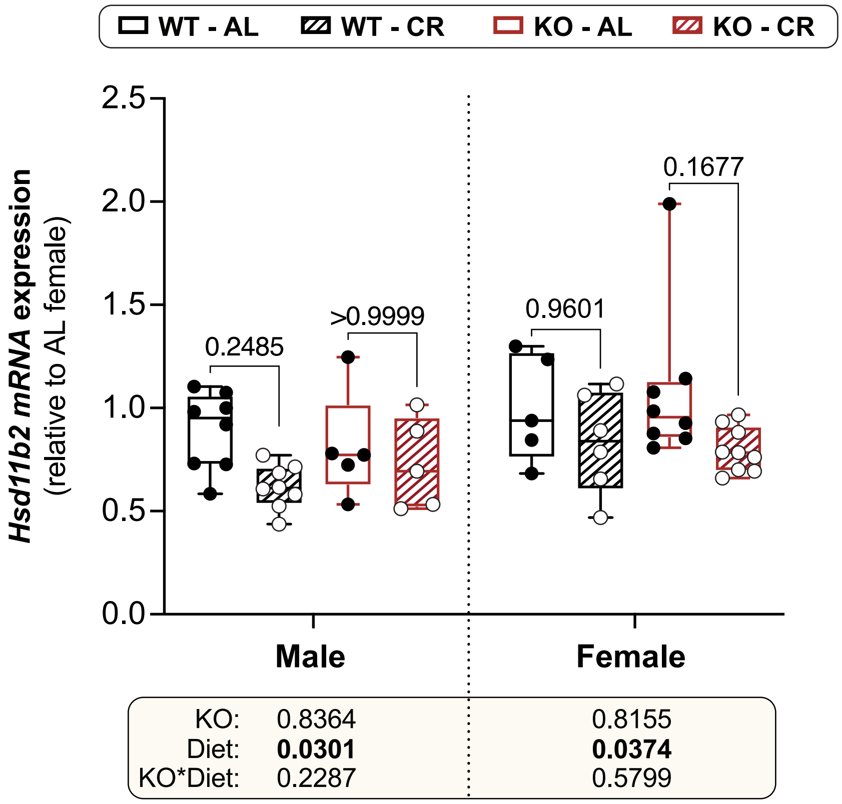

Supplementary Figure 5 – Effects of CR and *Hsd11b1* KO on mRNA expression of *Hsd11b2* in BM.
Male and female WT and *Hsd11b1* KO mice were fed AL or a 30% CR diet as described for Figure 2. Tibial BM was collected from 10-week-old mice at necropsy and *Hsd11b2* mRNA levels determined by qPCR. Expression is shown relative to levels in AL females after normalising to the geometric mean of the housekeeping genes *Ppia, Tbp* and *Actb*. Data presentation, numbers of mice per group, and statistical analyses are as described for Figure 7. Source data are provided as a Source Data file.

Supplementary Figure 6

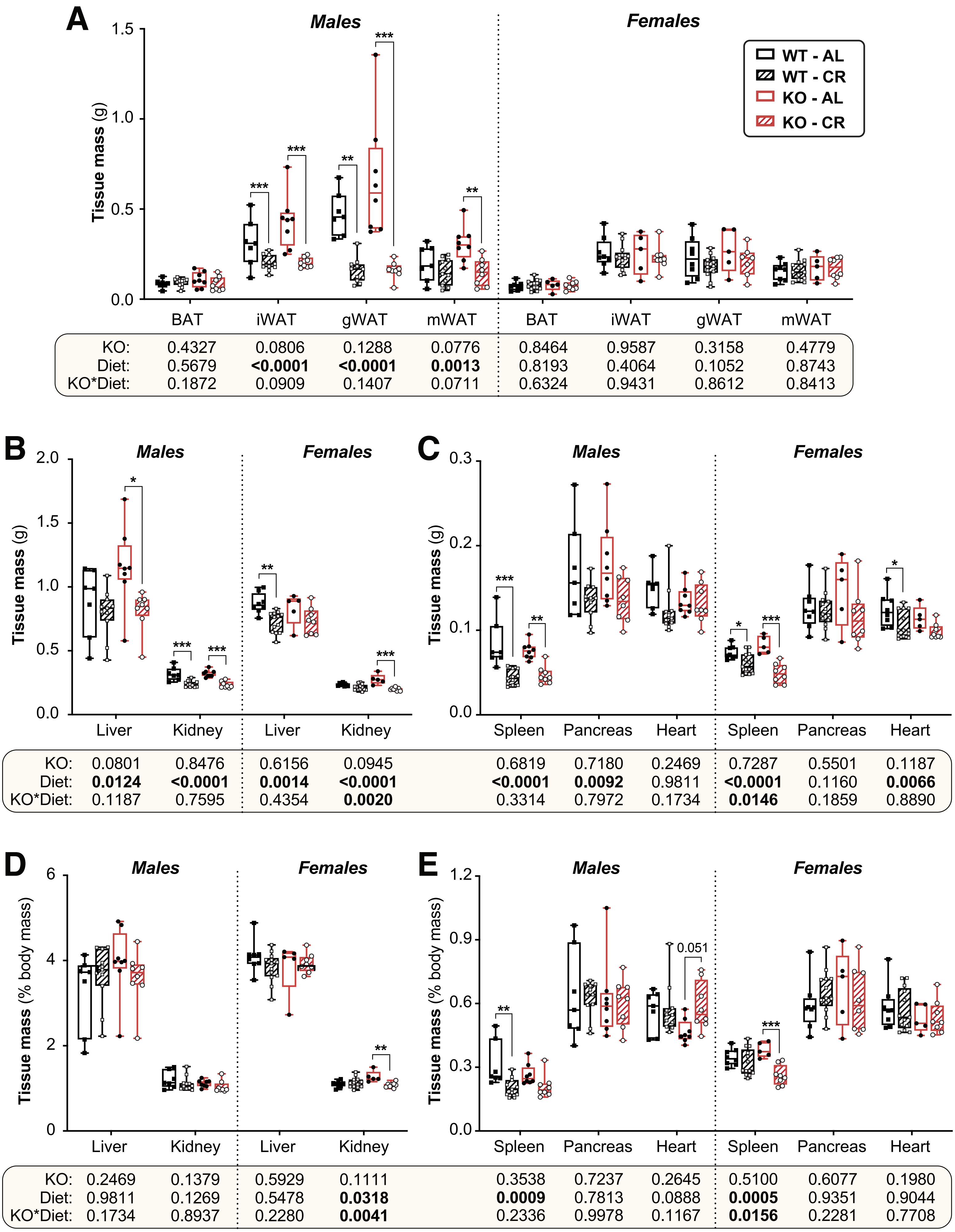

A

	Males BAT	Males iWAT	Males gWAT	Males mWAT	Females BAT	Females iWAT	Females gWAT	Females mWAT
KO:	0.4327	0.0806	0.1288	0.0776	0.8464	0.9587	0.3158	0.4779
Diet:	0.5679	**<0.0001**	**<0.0001**	**0.0013**	0.8193	0.4064	0.1052	0.8743
KO*Diet:	0.1872	0.0909	0.1407	0.0711	0.6324	0.9431	0.8612	0.8413

B

	Males Liver	Males Kidney	Females Liver	Females Kidney
KO:	0.0801	0.8476	0.6156	0.0945
Diet:	**0.0124**	**<0.0001**	**0.0014**	**<0.0001**
KO*Diet:	0.1187	0.7595	0.4354	**0.0020**

C

	Males Spleen	Males Pancreas	Males Heart	Females Spleen	Females Pancreas	Females Heart
KO:	0.6819	0.7180	0.2469	0.7287	0.5501	0.1187
Diet:	**<0.0001**	**0.0092**	0.9811	**<0.0001**	0.1160	**0.0066**
KO*Diet:	0.3314	0.7972	0.1734	**0.0146**	0.1859	0.8890

D

	Males Liver	Males Kidney	Females Liver	Females Kidney
KO:	0.2469	0.1379	0.5929	0.1111
Diet:	0.9811	0.1269	0.5478	**0.0318**
KO*Diet:	0.1734	0.8937	0.2280	**0.0041**

E

	Males Spleen	Males Pancreas	Males Heart	Females Spleen	Females Pancreas	Females Heart
KO:	0.3538	0.7237	0.2645	0.5100	0.6077	0.1980
Diet:	**0.0009**	0.7813	0.0888	**0.0005**	0.9351	0.9044
KO*Diet:	0.2336	0.9978	0.1167	**0.0156**	0.2281	0.7708

Supplementary Figure 6 – Effects of CR on tissue masses in WT and *Hsd11b1* KO mice
Male and female WT and *Hsd11b1* KO mice were fed AL or a 30% CR diet as described for Figure 2. **(A-C)** Absolute masses (g) of gWAT, iWAT, mWAT, BAT, the liver, kidneys, spleen, pancreas, and heart, as recorded at necropsy. **(D-E)** Masses of the liver, kidneys, spleen, pancreas, and heart, reported as % body mass. Data presentation and statistical analysis are as described for Figure 2G. Source data are provided as a Source Data file.
